# Motor hyperactivation during cognitive tasks: An endophenotype of juvenile myoclonic epilepsy

**DOI:** 10.1111/epi.16575

**Published:** 2020-06-25

**Authors:** Lorenzo Caciagli, Britta Wandschneider, Maria Centeno, Christian Vollmar, Sjoerd B. Vos, Karin Trimmel, Lili Long, Fenglai Xiao, Alexander J. Lowe, Meneka K. Sidhu, Pamela J. Thompson, Gavin P. Winston, John S. Duncan, Matthias J. Koepp

**Affiliations:** ^1^ Department of Clinical and Experimental Epilepsy UCL Queen Square Institute of Neurology London UK; ^2^ MRI Unit Epilepsy Society Chalfont St Peter Buckinghamshire UK; ^3^ Epilepsy Unit Hospital Clínic de Barcelona Barcelona Spain; ^4^ Department of Neurology Ludwig‐Maximilians‐Universität Munich Germany; ^5^ Centre for Medical Image Computing University College London London UK; ^6^ Neuroradiological Academic Unit UCL Queen Square Institute of Neurology London UK; ^7^ Department of Neurology Medical University of Vienna Vienna Austria; ^8^ Department of Neurology Xiangya Hospital of Central South University Changsha China; ^9^ Department of Neurology West China Hospital of Sichuan University Chengdu China; ^10^ Department of Neurology Queen's University Kingston ON Canada

**Keywords:** cognition, endophenotype, fMRI, juvenile myoclonic epilepsy, motor system

## Abstract

**Objective:**

Juvenile myoclonic epilepsy (JME) is the most common genetic generalized epilepsy syndrome. Myoclonus may relate to motor system hyperexcitability and can be provoked by cognitive activities. To aid genetic mapping in complex neuropsychiatric disorders, recent research has utilized imaging intermediate phenotypes (endophenotypes). Here, we aimed to (a) characterize activation profiles of the motor system during different cognitive tasks in patients with JME and their unaffected siblings, and (b) validate those as endophenotypes of JME.

**Methods:**

This prospective cross‐sectional investigation included 32 patients with JME, 12 unaffected siblings, and 26 controls, comparable for age, sex, handedness, language laterality, neuropsychological performance, and anxiety and depression scores. We investigated patterns of motor system activation during episodic memory encoding and verb generation functional magnetic resonance imaging (fMRI) tasks.

**Results:**

During both tasks, patients and unaffected siblings showed increased activation of motor system areas compared to controls. Effects were more prominent during memory encoding, which entailed hand motion via joystick responses. Subgroup analyses identified stronger activation of the motor cortex in JME patients with ongoing seizures compared to seizure‐free patients. Receiver‐operating characteristic curves, based on measures of motor activation, accurately discriminated both patients with JME and their siblings from healthy controls (area under the curve: 0.75 and 0.77, for JME and a combined patient‐sibling group against controls, respectively; *P* < .005).

**Significance:**

Motor system hyperactivation represents a cognitive, domain‐independent endophenotype of JME. We propose measures of motor system activation as quantitative traits for future genetic imaging studies in this syndrome.

**Key Points**

- Both patients with juvenile myoclonic epilepsy (JME) and their unaffected siblings show enhanced activation of the motor system during memory and language functional magnetic resonance imaging (fMRI).
- Among patients, effects are more marked in those with ongoing seizures compared to those seizure‐free, suggesting modulation by disease activity.
- Measures of motor activation achieve accurate individual discrimination of patients with JME and their siblings from controls.
- Motor system hyperactivation is validated as a cognitive, domain‐independent endophenotype of JME.

## 1 INTRODUCTION

Juvenile myoclonic epilepsy (JME) is a common genetic generalized epilepsy (GGE) syndrome with a polygenetic etiology.^1^ Myoclonic jerks are its defining feature, and may be triggered by cognitive tasks, particularly those involving motor responses, ideation or execution of motor sequences, and language‐related activities.^2^ Neuropsychological assessments detected predominantly impaired frontal lobe function.^3^ Abnormal fronto‐thalamo‐cortical circuitry^4^, ^5^ and increased motor to prefrontal connectivity^6^ are likely neural correlates of cognitive dysfunction and ictogenesis.

In disorders with high heritability, such as JME, recent investigations have assessed neurobehavioral traits in unaffected siblings. This approach controls for potential influences of medication, interictal epileptiform discharges, and seizure load, and allows testing of individuals with comparable age, upbringing, and socioeconomic status. Although the investigation of siblings does not disentangle effects related to disease variables in patients, it provides the opportunity to capture common patterns in both disease‐affected and unaffected family members. These can be conceptualized as features of the disease that are independent of seizure activity or medication, and qualify as intermediate phenotypes or *endophenotypes*, that is, heritable traits, co‐segregating in families with affected members, and related to pathological mechanisms.^7^

Imaging genetics investigations ascertain the genetic underpinnings of brain structure and function.^8^ In disorders with multifactorial etiology, such as the epilepsies, imaging endophenotypes provide a link between clinical features and the underlying genetic architecture, informing on more proximal mechanisms that mediate neural systems‐level phenomena, and facilitating the identification of disease‐relevant genetic variants.^9^, ^10^ Endophenotype research is established in psychiatry,^9^ and has recently been applied to epilepsy. In temporal lobe epilepsy, family studies detected overlapping alterations of temporal cortical surface area,^11^, ^12^ absence of shared thickness abnormalities,^13^ and varying patterns of hippocampal atrophy.^11^, ^14^, ^15^ In GGE, dysexecutive traits appear common to patients and first‐degree relatives.^16^ Impairment of prospective memory and executive functions has been noted in patients with JME and unaffected siblings,^17^, ^18^ along with shared patterns of frontocortical morphometric abnormalities,^19^ hippocampal volume loss, and malpositioning.^20^ Myoclonus is a pathognomonic feature of JME, and recent imaging research has focused on the motor system. In a proof‐of‐concept study series, we detected co‐activation of motor and frontoparietal cognitive areas during a complex visuospatial working memory task both in patients with JME and their siblings.^21^, ^22^ These findings supported the hypothesis that patterns of motor activity during executive demand in JME may be non‐normative, and likely heritable.

In this study, we aimed to advance prior work, and deliver a quantitative validation of motor system hyperactivation across cognitive domains as an endophenotype of JME. We compared patterns of functional magnetic resonance imaging (fMRI) activation in patients with JME, their unaffected siblings, and healthy controls during two common cognitive paradigms that recruit motor areas and address episodic memory encoding and expressive language. These are higher cognitive functions often affected in epilepsy, and can be readily assessed in most tertiary epilepsy centers via clinically established imaging protocols.^23^ To further ascertain the endophenotypic potential of motor system activation, we also probed individual discrimination of patients and siblings from controls. Positive results would motivate the use of motor activation metrics as quantitative markers to dissect the underlying genetic and molecular mechanisms of motor hyperexcitability, and identify genetic variants predisposing to JME.

## 2 METHODS

### 2.1 Participants

For this prospective cross‐sectional investigation, we consecutively recruited 32 patients with JME, 12 unaffected siblings of 11 index patients, and 26 healthy controls with no family history of epilepsy, comparable for age, sex, and handedness, between 2007 and 2013 (Table 1).

**TABLE 1:** Demographic details, neuropsychological test results, task performance, and motor system laterality indices

	JME	SIB	CTR	Test statistic	*P*‐value
Age at scan (y)	32.0 (16)	41.5 (25)	30.5 (9)	1.64^a^	.44
Sex (F/M)	17/15	8/4	15/11	0.66^b^	.72
Handedness (L/R)	2/30	1/11	2/24	0.46^b^	1.00
Time of MRI acquisition (h)	11 (4)	15 (1)	11 (4.3)	6.62^a^	.04
*JME vs CTR*				−0.31	1.00^c^
*SIB vs CTR*				−2.19	.085^c^
*JME vs SIB*				−2.5	.037^c^
Age at disease onset (y)	15 (4.3)	N/A	N/A	N/A	N/A
Disease duration (y)	16.5 (17.5)	N/A	N/A	N/A	N/A
Time since last seizure (y)	1.0 (3.9)	N/A	N/A	N/A	N/A
AEDs at time of scan (number)	2 (1)	N/A	N/A	N/A	N/A
HADS/Anxiety	6 (3)	5 (2)	4 (5)	3.60^a^	.17
HADS/Depression	2 (4)	1 (2)	1.5 (1)	3.37^a^	.19
Language LI (frontal)	0.73 (0.3)	0.79 (0.2)	0.64 (0.2)	1.80^a^	.41
Memory LI, Words (frontal)	0.59 (0.3)	0.53 (0.2)	0.56 (0.4)	0.63^a^	.73
NART IQ	111.0 (10.5)	106.0 (17.5)	113.0 (9.0)	2.27^a^	.32
Letter fluency	44.0 (19.8)	44.50 (9.8)	46.0 (17.8)	1.69^a^	.43
Category fluency	54.0 (21.0)	53.5 (14.5)	53.0 (8.0)	1.33^a^	.51
List learning (A1‐A5)	58.0 (16.0)	57.50 (12.8)	56.0 (10.5)	1.74^a^	.42
List learning (A6)	12.0 (5.0)	12.0 (3.0)	12.0 (4.0)	0.20^a^	.91
Design Learning (A1‐A5)	38.0 (13.0)	38.5 (8.8)	37.0 (13.5)	1.68^a^	.51
Design learning (A6)	8.0 (3.0)	9.0 (1.0)	9.0 (2.0)	6.26^a^	.19
Trail Making Test A	29.0 (9.0)	24.00 (13.8)	31.0 (15.0)	2.05^a^	.36
Trail Making Test B‐A	32.0 (20.0)	21.0 (15.0)	22.5 (17.8)	7.75^a^	.02^d^
*JME vs CTR*				2.41	.047^c^
*SIB vs CTR*				−0.18	1.00^c^
*JME vs SIB*				2.02	.132^c^
Digit span	19.0 (7.0)	20.0 (4.0)	19.0 (4.8)	1.49^a^	.48
Mental arithmetic	14.0 (7.3)	13.0 (5.0)	17.0 (6.0)	2.40^a^	.30
Motor LI—Language fMRI, “Repeat”	0.37 (0.7)	0.42 (0.5)	0.20 (0.6)	2.07^a^	.36
Motor LI—Language fMRI, “Generate”	0.67 (0.4)	0.73 (0.2)	0.62 (0.4)	2.49^a^	.29
Motor LI – Memory fMRI, “Pictures”	0.65 (0.3)	0.76 (0.3)	0.76 (0.3)	2.55^a^	.28
Motor LI—Memory fMRI, “Words”	0.73 (0.3)	0.67 (0.5)	0.79 (0.2)	1.43^a^	.49
Motor LI—Memory fMRI, “Faces”	0.56 (0.7)	0.54 (0.4)	0.7 (0.5)	1.92^a^	.38
Reaction time—Memory fMRI (s)	1.27 (0.2)	1.35 (0.2)	1.32 (0.2)	3.11^a^	.21
Response Rate—Memory fMRI (%)	99.3 (1.4)	99.5 (2.0)	99.5 (2.4)	0.44^a^	.81
Recognition Accuracy—Memory fMRI, “Pictures” (%)	82.9 (17.1)	84.3 (12.1)	86.4 (23.2)	0.61^a^	.74
Recognition Accuracy—Memory fMRI, “Words” (%)	80.0 (19.3)	80.0 (22.5)	84.3 (35.7)	0.40^a^	.82
Recognition Accuracy—Memory fMRI, “Faces” (%)	27.1 (17.9)	27.9 (20.7)	37.1 (25.7)	3.68^a^	.16

Note

Continuous variables are reported as median (interquartile range). Neuropsychological test scores are reported as raw. Reaction times for the memory fMRI task are reported in seconds, and correspond to the average time interval between item display and associated joystick response. "Response rate" refers to the proportion of actual joystick responses in relation to the total number of possible responses (i.e., tracks missed responses and task compliance).

Abbreviations: AED, anti‐epileptic drug; CTR, healthy controls; HADS, Hospital Anxiety and Depression Scale; JME, juvenile myoclonic epilepsy; LI, laterality index; NART, National Adult Reading Test; SIB, siblings of patients with JME.

^a^ Kruskal‐Wallis test, *H* statistic.

^b^ Fisher's exact test, chi‐square statistic.

^c^ Bonferroni‐corrected post hoc tests, with accompanying standardized test statistics.

^d^ *P*‐value not surviving correction for multiple comparisons across cognitive measures.

Patients with JME were recruited from outpatient clinics at the National Hospital for Neurology and Neurosurgery, London, and the Epilepsy Society, Buckinghamshire. Siblings were recruited through index patients. Controls were recruited from the local community. All patients had a typical history of JME, with onset of myoclonic jerks and generalized tonic‐clonic seizures during adolescence; 14 of 32 (44%) had absences. Scalp electroencephalography (EEG) showed generalized polyspike‐wave discharges, and routine brain MRI scans were normal. Further clinical details are available in Appendix S1, Table 1 and Table S1. Fifty‐three percent of the patients had been seizure‐free for longer than a year before the investigation. Siblings and controls had never experienced unprovoked seizures. One sibling had had two clearly provoked generalized tonic‐clonic seizures >20 years before study participation, owing to alcohol intoxication and sleep deprivation, with normal investigations, without any further seizures, and without anti‐epileptic medication.

### 2.2 Standard protocol approvals, registrations, and patient consents

The study was approved by the London South‐East Research Ethics Committee and by the University College London and University College London Hospitals Joint Research Office. Written informed consent was obtained from all participants.

### 2.3 Neuropsychological data

Participants completed handedness, depression, and anxiety questionnaires. Neuropsychological tests (Table 1) provided measures of estimated intellectual level (National Adult Reading Test),^24^ verbal generativity, verbal and visual learning, working memory, psychomotor speed, and mental flexibility. Test descriptions are provided in Appendix S1. Response rates and reaction times during the memory fMRI task were recorded, and may represent additional measures of attention and processing speed. However, these metrics could also relate to task accuracy and strategy, item difficulty, and participants' confidence in individual responses.

### 2.4 MRI data acquisition and fMRI paradigms

Functional MRI data were acquired for all participants on a 3T GE Signa‐HDx MRI scanner using a previously described sequence.^25^ All data were acquired between 10:00 and 17:00 hours. In view of the circadian dependency of myoclonus in JME, time of MRI acquisition was recorded and compared across groups.

During the memory task, 10 pictures, 10 words, and 10 faces were presented every 3 seconds within 30 second blocks, separated by 15 second cross‐hair fixation^26^ (total of 210 items; 70 pictures/words/faces). Participants were instructed to memorize the items for subsequent out‐of‐scanner recall, while using a joystick for a subjective decision on stimulus pleasantness. All participants operated the joystick with their right hand. Reaction times and response rates were recorded. The memory task also entailed an out‐of‐scanner recall session, during which the previously presented items were intermixed with an additional 50% novel stimuli. Recognition accuracy was calculated as true positive rate minus false positive rate.^26^ Memory fMRI data were available for 28 patients, all siblings, and 20 controls. The expressive language task consisted of 30 second blocks, during which subjects were asked to covertly generate verbs associated with a visually displayed noun (“Generate” condition), or to repeat a visually displayed noun (“Repeat” condition). There were six blocks for each condition, intermixed with six cross‐hair fixation blocks.^27^ Verb generation fMRI data were acquired in all participants.

### 2.5 Data analysis

Clinical and neuropsychological data were analyzed with SPSS Statistics 24.0, using chi‐square and Kruskal‐Wallis tests for categorical and continuous non‐parametric data, respectively. Imaging data were preprocessed with Statistical Parametric Mapping 8 (SPM8) as previously described (Appendix S1).^25^

A two‐level random‐effects analysis was employed. At the first level, condition‐specific effects were estimated according to the general linear model for each subject. Task conditions were modeled separately as 30 second blocks and convolved with the canonical hemodynamic response function. Individual‐level contrasts were created to detect activation associated with encoding pictures, words, and faces against baseline, and with the “Repeat” and “Generate” conditions against baseline. Parameter estimates were calculated voxel‐wise for each regressors, and six motion parameters were included as regressors of no interest.

Laterality indices (LIs) assessing frontal hemispheric dominance for verbal processing were computed based on the word‐generation language contrast, and the word‐encoding contrast of the memory task, using the bootstrap method and a bilateral frontal lobe mask of the SPM LI toolbox.^28^ In addition, LIs of motor system activation were computed for each condition of both fMRI tasks in each subject, using a bilateral functional motor system mask (motor region of interest [ROI]), consisting of parcels 9‐53‐55‐57‐61 of the *Brainnetome* Atlas^29^ and their homologous right counterparts, comprising bilateral precentral gyrus and supplementary motor area. Because the verb generation fMRI paradigm was conducted covertly, performance measures during task execution were not available. All maps were reviewed for activation of expressive language‐relevant areas (inferior frontal and middle frontal gyrus) up to a threshold of *P* < .01, uncorrected.^25^ One patient was excluded because of lack of activation.

For group‐level analyses, one‐sample *t* tests assessed the effect of each condition across all subjects. Conjunction analyses^30^ illustrated common significant activations across conditions for each task. Peak‐level activations were considered statistically significant at a threshold of *P* < .05, controlling for family‐wise error (FWE) rate. Group comparisons were conducted using full factorial designs, with (a) group and (b) item category/condition type as factors, to identify intergroup differences (1) across item categories for the memory task, (2) across conditions for the language task, as well as (3) across all memory and language conditions altogether, via a “combined task” model. In accord with standard SPM procedures, intergroup differences were first identified via *F* tests, which were significant for all tasks and models (Tables [Link], [Link], [Link]). Pairwise comparisons addressed greater activation in patients than controls, and siblings than controls, via *t* contrasts. For completeness, decreased activation in patients and siblings vs controls was assessed via reversed SPM contrasts. As previously,^22^ we employed conjunction analyses^30^ of patients greater than controls and siblings greater than controls to further substantiate areas of common intergroup differences in activation magnitude. For all task models, subgroup analyses compared JME patients with ongoing seizures against those seizure‐free and controls. We also repeated all group comparisons using age, sex, and handedness as confound regressors. For anatomical localization purposes, we used the above‐described motor ROI. In view of our a priori hypothesis, peak‐level group differences in motor activation were considered statistically significant at *P* < .05, FWE‐corrected within a 12 mm diameter sphere (small volume) centered at the location of the maxima in the motor ROI (FWE‐svc).^31^, ^32^ The remainder whole‐brain effects are reported at an exploratory threshold of *P* < .005 uncorrected, with a 20‐voxel minimum cluster‐size threshold.^33^, ^34^

### 2.6 Group discrimination via measures of motor system activation

To complement validation of measures of motor system activation as JME endophenotype, we employed those within receiver‐ operating characteristic (ROC) curve analyses, and assessed their accuracy in discriminating JME patients and their siblings from healthy controls at the individual level. Initial models evaluated discrimination via motor activation during memory and language tasks separately, and implemented age‐, sex‐, and handedness‐adjusted contrast estimates (beta weights) of task‐related activation extracted from areas of common intergroup differences in each task, as determined via conjunction analyses. Specifically, individual contrast estimates were extracted from peaks located in the *left* precentral gyrus, for both language and memory tasks (Montreal Neurological Institute [MNI] coordinates are provided in Tables S2 and S3); right‐sided motor areas did not represent common areas of increased activation in patients and siblings, and were not considered. Additional ROC curve models relied on a composite marker of cognition‐related motor activation. The latter was obtained after extracting and averaging individual contrast estimates across all language and memory conditions from the area of common intergroup difference detected via conjunction analysis on the “combined task” fMRI model, located in the *left* precentral gyrus (see Table S4 for peak MNI coordinates). For all three measures (motor activity for memory, language, and combined model), we investigated (a) discrimination of patients with JME from healthy controls, and (b) discrimination of a combined group of patients and their unaffected siblings from healthy controls, via the area under the curve (AUC) metric.^20^

## 3 RESULTS

### 3.1 Demographics and neuropsychological data

There were no intergroup differences for age, sex, handedness, anxiety and depression scores, reaction times, response rate, or recognition accuracy during the memory task (all *P* > .05; Table 1). Time of MRI acquisition differed across groups (*P* = .04, Table 1; sensitivity analyses detailed in Section 3.7, "Sensitivity analyses: influence of time of MRI acquisition"). There were no significant differences in time of MRI acquisition between patient subgroups with and without ongoing seizures (median [interquartile range, IQR]: 11.0/11.0 (4.0/2.5), respectively; Mann‐Whitney *U* test, *P* = .28).

Comparison of cognitive scores showed between‐group differences in mental flexibility (*P* = .02), with post hoc tests indicating worse performance in patients with JME than controls (*P* = .047, Bonferroni‐corrected), and no differences between patients and siblings (Table 1). The latter did not survive correction for multiple comparisons across cognitive tests (false discovery rate procedure, adjusted *P*‐value: .22). There were no other differences in neuropsychological test scores.

### 3.2 Task performance and laterality indices

There were no intergroup differences in reaction times, response rate, or recognition accuracy during the memory task (all *P*’s > .16, Table 1). Frontal lobe LIs for expressive language and verbal encoding did not differ across groups (all *P*’s > .41, Table 1), indicating similar patterns of lobar hemispheric dominance for verbal processing. There were no group differences for laterality indices of motor system activation for all language and memory task conditions (all *P*’s > .28, Table 1).

### 3.3 Memory encoding fMRI task

**FIGURE 1:**
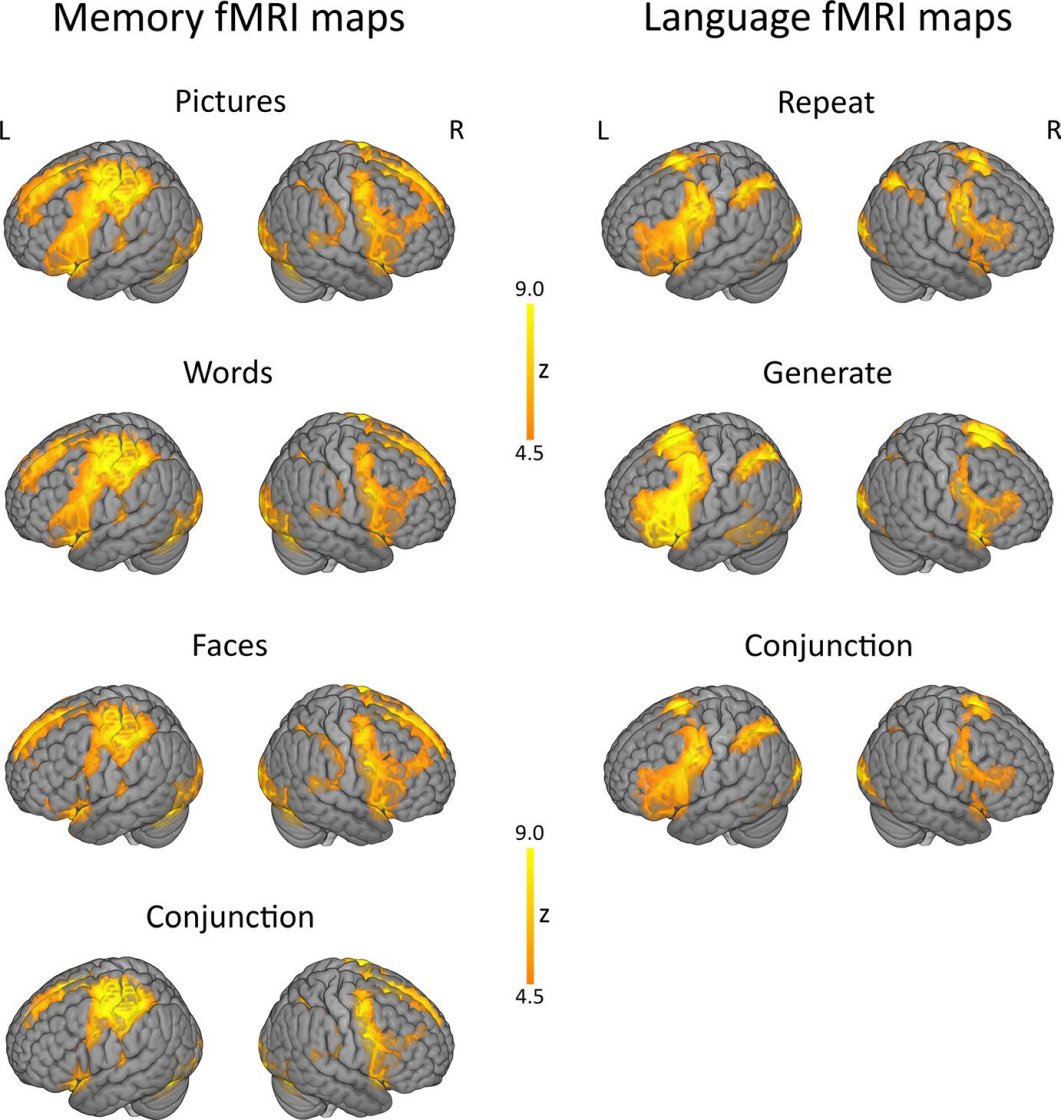
Activation maps for memory and language functional magnetic resonance imaging (fMRI). The figure shows whole‐brain activation maps, obtained via one‐sample *t* tests across all subjects, for the effect of encoding pictures, words, and faces (left‐hand side) during the memory fMRI task, and for word repetition and generation during the expressive language fMRI task (right‐hand side). Memory‐associated activation in frontal lobe areas is left lateralized for word encoding, bilateral for picture encoding, and right lateralized for face encoding. Fronto‐temporo‐parietal language fMRI activation is left lateralized, and effects in the left middle and inferior temporal gyrus are more marked during the verb generation condition. Conjunction analyses represent multi‐dimensional equivalent of one‐sided *t* tests,^30^ and highlight consistent effects across task conditions. All activation maps are thresholded at *P* < .05, family‐wise error (FWE) corrected for multiple comparisons across the whole brain

The task activated bilateral motor and premotor areas for all stimulus types, with left‐sided predominance. Effects in prefrontal and temporoparietal cortices appeared material‐specific (Figure 1), being more left lateralized for words, right lateralized for faces, and intermediate for pictures, which are visually presented but may be verbalized during encoding.

**FIGURE 2:**
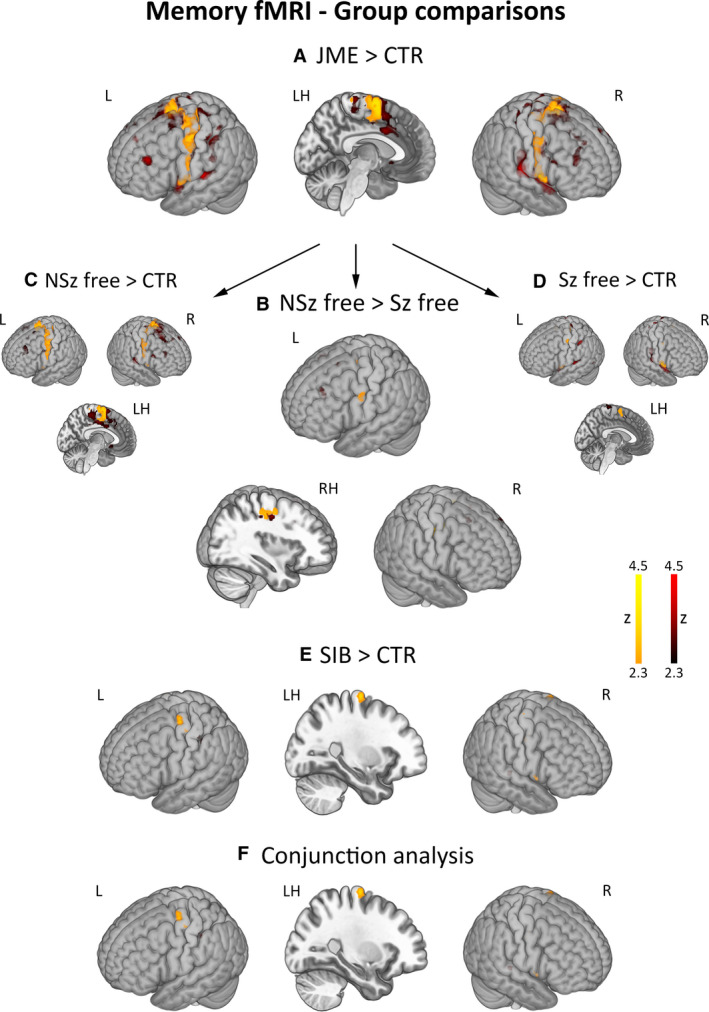
Group comparisons for memory fMRI activation. Across item categories, the figure shows areas of enhanced activation for comparisons of patients with juvenile myoclonic epilepsy (JME) against controls (JME > CTR, panel A), patients with ongoing seizures against those seizure‐free (NSz free > Sz free, panel B), and JME siblings against controls (SIB > CTR, panel E). Comparison of (a) JME patients with ongoing seizures against controls and (b) seizure‐free JME patients against controls is also provided for completeness (panels C and D, respectively). Conjunction analysis^30^ identified shared areas of hyperactivation in patients and siblings (panel F). Comparisons for motor system and remainder whole‐brain effects are shown with different color scales (*orange‐yellow* scale for motor regions, *red* scale for the remainder brain areas). *P*‐values for activation differences within the motor system were corrected for family‐wise error rate using 12‐mm diameter spherical regions of interest centered on local maxima. “LH/RH” refer to sagittal sections of the left/right hemisphere. Color bars reflect *z*‐score scales. MNI coordinates and statistical details are provided in Table S2

Across item categories, patients with JME exhibited increased activation of the supplementary motor area (SMA), and both patients with JME and siblings showed hyperactivation of the motor cortex compared with controls. Conjunction analysis of higher activation in patients and siblings than controls identified common significant effects within the left motor cortex. Subgroup analyses showed more prominent motor activation in JME patients with ongoing seizures compared to seizure‐free patients; enhanced activation of the motor system was independently confirmed for both JME subgroups (Figure 2). Post hoc Spearman's correlations substantiated a negative association between time since last seizure and left precentral activation (*ρ* = −0.39, *P* = .04; Appendix S1). For all group comparisons, repeat models using age, sex, and handedness as covariates provided virtually identical results (Table S2, Appendix S1). Post hoc correlations found no significant associations between motor activation estimates in the area of common group differences and (a) verbal memory LI as well as (b) motor LIs for all memory task conditions (Appendix S1).

Exploratory whole‐brain analyses identified higher activation of bilateral fronto‐temporo‐parietal cortices in patients with JME compared to controls, and higher activation of the left prefrontal and bilateral cingulate cortices in patients with ongoing seizures compared to those seizure‐free (Figure 2, Table S2). At uncorrected thresholds, siblings exhibited lower activation of the right lingual gyrus. There were no areas of decreased activation in patients with JME compared to controls, and in patients with ongoing seizures compared to those seizure‐free.

### 3.4 Language fMRI task

The task activated motor cortex, SMA, inferior parietal lobule, and middle and inferior frontal gyrus during both “Repeat” and “Generate” conditions, and lateral temporal cortices for “Generate” only, with left‐sided predominance (Figure 1).

**FIGURE 3:**
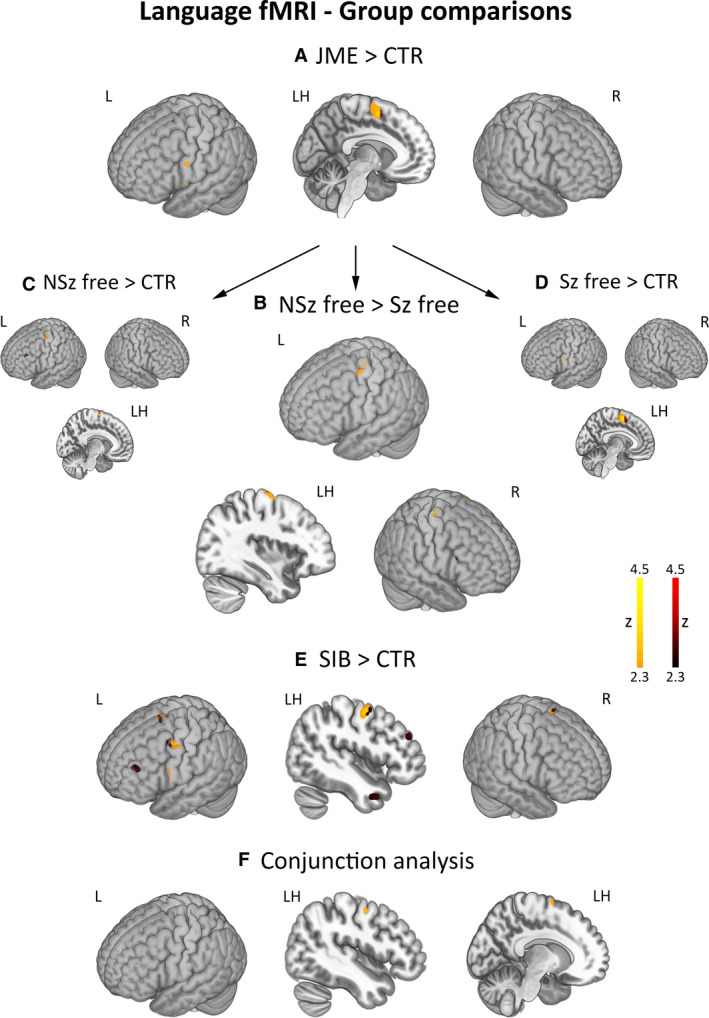
Group comparisons for language functional magnetic resonance imaging (fMRI) activation. Across language task conditions, the figure displays areas of enhanced activation for comparisons of patients with juvenile myoclonic epilepsy (JME) against controls (JME > CTR, panel A), patients with ongoing seizures against those seizure‐free (NSz free > Sz free, panel B), and JME siblings against controls (SIB > CTR, panel E). Comparison of (a) JME patients with ongoing seizures against controls and (b) seizure‐free JME patients against controls are also provided for completeness (panels C and D, respectively). Conjunction analysis^30^ identified shared areas of hyperactivation in patients and siblings (panel F). Comparisons for motor system and remainder whole‐brain effects are shown with different color scales (*orange‐yellow* scale for motor regions, *red* scale for the remainder brain areas). *P*‐values for motor system activation differences were corrected for family‐wise error rate using 12‐mm diameter spherical regions of interest centered on local maxima. “LH” refers to a sagittal section of the left hemisphere. Color bars reflect z‐score scales. Montreal Neurological Institute (MNI) coordinates and statistical details are provided in Table S3

Across conditions, patients with JME and their unaffected siblings had greater activation of the motor cortex and SMA than controls. Conjunction analysis of stronger activation in JME and siblings than controls identified significant motor system effects. Subgroup analyses showed more marked motor activation in patients with ongoing seizures compared to seizure‐free individuals; increased activation of the motor system was independently confirmed for both JME subgroups (Figure 3); post hoc Spearman's correlations showed a nonsignificant, negative association between time since last seizure and left precentral activation (*ρ* = −0.22, *P* = .24; Appendix S1). For all group comparisons, repeat models using age, sex, and handedness as covariates provided virtually identical results (Table S3, Appendix S1). Post hoc correlations found no significant associations between motor activation estimates in the area of common group differences and (a) language LI as well as (b) motor LIs for all language task conditions (Appendix S1).

Exploratory whole‐brain analyses showed higher activation of the left middle frontal gyrus in JME patients with ongoing seizures against those seizure‐free, and stronger activation of left middle frontal gyrus in siblings compared to controls.

There were no areas of decreased activation in patients with JME and siblings compared to controls, and for the comparison of patients with ongoing seizures against those seizure‐free (Figure 3, Table S3).

### 3.5 Combined fMRI activation model across all conditions

Group comparisons across tasks showed increased activation of the SMA in JME, and hyperactivation of the motor cortex in both patients with JME and unaffected siblings compared to controls. Conjunction analysis of higher activation in patients and siblings than controls identified common significant effects within the left motor cortex. Motor activation was more prominent in JME patients with ongoing seizures compared to seizure‐free patients; enhanced activation of the motor system was independently confirmed both for JME subgroup. Post hoc Spearman's correlations showed a strong negative association between time since last seizure and left precentral activation (*ρ* = −0.55, *P* = .005; Appendix S1). For all group comparisons, repeat models using age, sex, and handedness as covariates provided virtually identical results (Table S4, Appendix S1).

**FIGURE 4:**
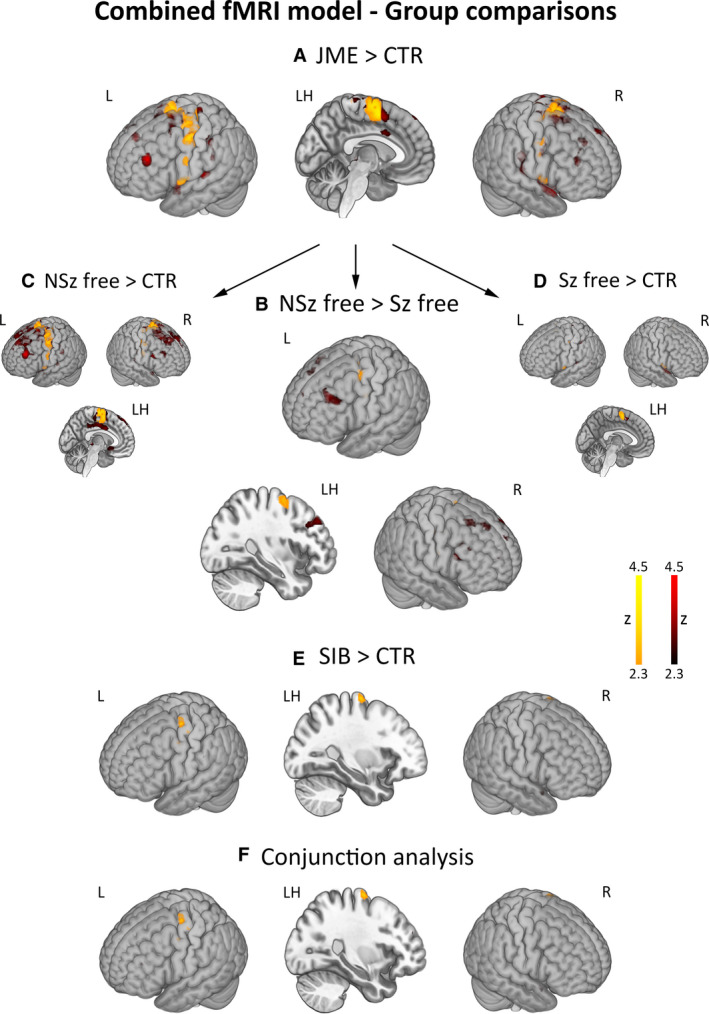
Group comparisons for combined activation model across functional magnetic resonance imaging (fMRI) tasks. Pooling across all memory and language conditions, the figure displays areas of enhanced activation for comparisons of patients with juvenile myoclonic epilepsy (JME) against controls (JME > CTR, panel A), patients with ongoing seizures against those seizure‐free (NSz free > Sz free, panel B), and JME siblings against controls (SIB > CTR, panel E). Comparison of (a) JME patients with ongoing seizures against controls and (b) seizure‐free JME patients against controls are also provided for completeness (panels C and D, respectively). Conjunction analysis^30^ identified shared areas of hyperactivation in patients and siblings (panel F). Comparisons for motor and remainder whole‐brain effects are shown with different color scales (*orange‐yellow* scale for motor regions, *red* scale for the remainder brain areas). *P*‐values for motor system activation differences were corrected for family‐wise error rate using 12‐mm diameter spherical regions of interest centered on local maxima. “LH” refers to a sagittal section of the left hemisphere. Color bars reflect *z*‐score scales. MNI coordinates and statistical details are provided in Table S4

Exploratory whole‐brain analyses detected higher activation of bilateral fronto‐temporo‐parietal areas in JME compared to controls, and more marked activation of prefrontal and cingulate cortices in patients with ongoing seizures against those seizure‐free. There were no areas of decreased activation in patients with JME and their siblings compared to controls, and in patients with ongoing seizures compared to those seizure‐free (Figure 4, Table S4).

### 3.6 ROC curve analyses

**FIGURE 5:**
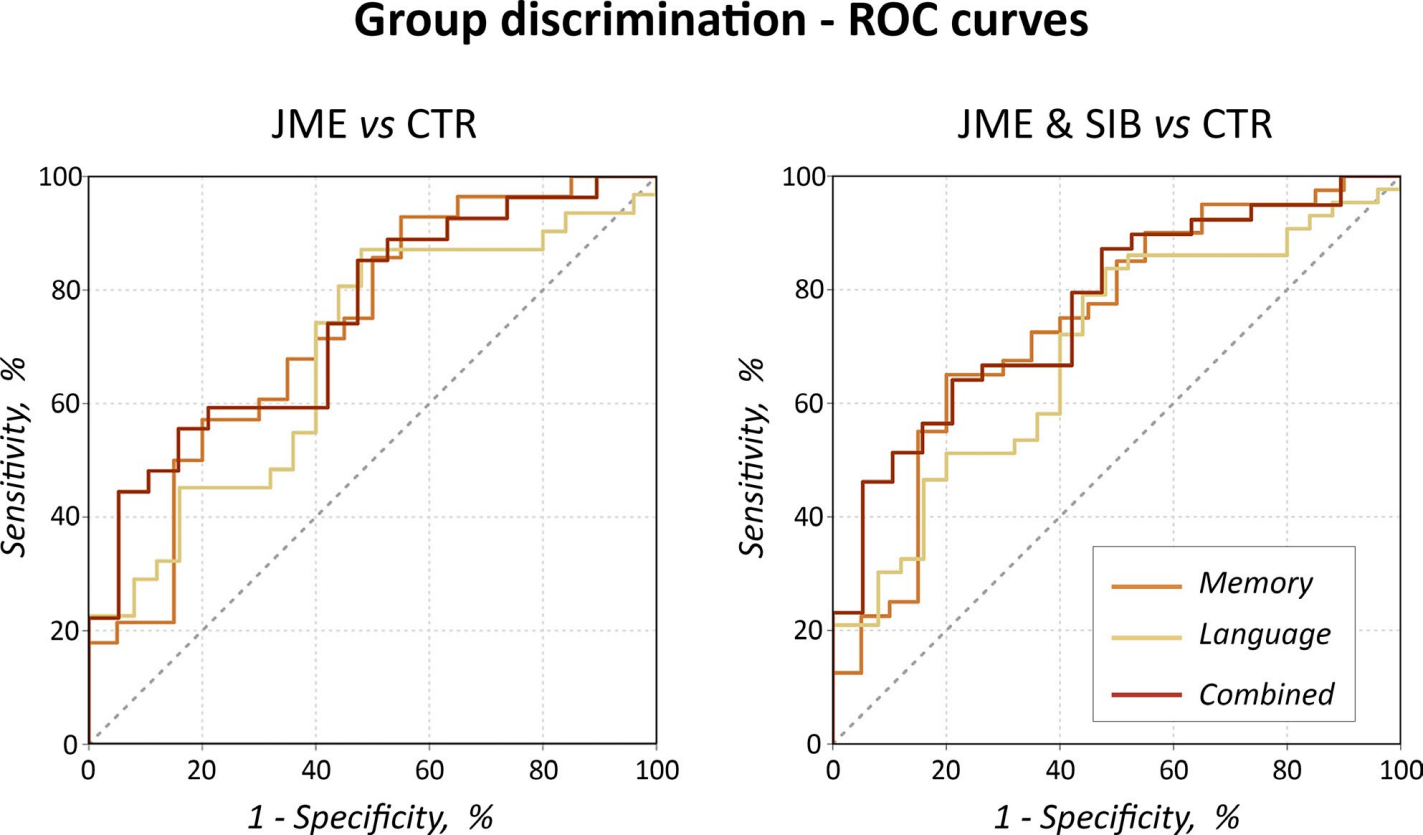
Receiver‐operating characteristic (ROC) curve analyses on measures of motor activation. The figure shows ROC curves probing usefulness of motor system activation metrics in discriminating patients with juvenile myoclonic epilepsy (JME) from controls (left panel), and JME patients and their siblings, considered as a unitary group, from controls (right panel). Separate analyses were conducted for motor activation during memory and language functional magnetic resonance imaging (fMRI) tasks (orange and beige curves, respectively), whereas a third analysis employed parameter estimates derived from a combined fMRI model, averaging across all cognitive conditions (dark red curve). Individual discrimination of both JME patients and siblings from controls was significant in all analyses (all *P* < .02), with measures derived from the composite model leading to slightly higher classification accuracy (area under the curve [AUC] 0.75/0.77, for individual discrimination of JME/combined JME‐sibling group from controls, respectively; both *P* < .005). Full statistical details are provided in section 3.6

ROC curve analyses (Figure 5) highlighted successful discrimination of patients with JME and healthy controls via measures of motor system activation, both during memory and language tasks, with superior accuracy of the former (memory fMRI: AUC = 0.73, standard error [SE] = 0.07, *P* = .007; language fMRI: AUC = 0.68, SE = 0.07, *P* = .019). Use of a composite marker of motor activation across tasks slightly improved classification accuracy (AUC = 0.75, SE = 0.07, *P* = .004). The above models were repeated after combining patients with JME and their unaffected siblings into a unitary group. Again, ROC curve analyses documented successful individual discrimination, both with metrics derived from memory and language tasks separately (memory fMRI: AUC = 0.74, SE = 0.07, *P* = .003; language fMRI: AUC = 0.69, SE = 0.07, *P* = .009), as well as based on the composite marker of motor activity, which yielded improved classification accuracy (AUC = 0.77, SE = 0.06, *P* = .001).

Hence, these analyses point to relatively high discrimination of both JME patients and a combined JME‐sibling group from healthy controls, indicating co‐segregation of cognition‐related motor system hyperactivation in patients and their unaffected relatives, and further corroborating its endophenotypic potential.

### 3.7 Sensitivity analyses: influence of time of MRI acquisition

Time of day of MRI acquisition differed across groups, with JME and controls being predominantly scanned late in the morning, and siblings in the early afternoon hours. Corrected post hoc tests showed significant differences between JME and siblings, and trend‐level *P*‐values for comparison of siblings and controls (Table 1). We thus devised the following sensitivity analyses, described in Appendix S1: (a) rank correlations between time of day and metrics of motor system activation across participants, controlling for the effect of group; (b) repeat second‐level fMRI models for groups exhibiting significant/near‐significant differences in time of MRI acquisition, using the latter as covariate; (c) repeat ROC curve analyses employing contrast estimates of motor activation adjusted for age, sex, handedness, as well as time of MRI. These analyses produced largely similar results to the main analyses, pointing to a marginal influence of time of MRI acquisition on group‐specific patterns of cognition‐related motor activation.

## 4 DISCUSSION

Using two fMRI paradigms, we detected enhanced cognition‐related motor system activation in patients with JME and their unaffected siblings, and validated the latter as a robust functional intermediate phenotype (endophenotype) of JME.

Myoclonic jerks represent the hallmark of JME. Neurophysiological investigations have implicated a frontocortical generator,^35^, ^36^ and transcranial magnetic stimulation studies have indicated motor cortex hyperexcitability in patients and their siblings,^37^ suggesting trait heritability. In JME, myoclonus may be precipitated by cognitive activities, including reading, decision‐making, and ideation or execution of complex motor sequences.^2^ Our group previously reported co‐activation of motor and cognitive areas in patients and their siblings during a complex visuospatial working memory task, suggesting altered activity profiles of the motor system during executive demands.^21^, ^22^

Here, we challenged the reactivity of the motor system with two previously unreported cognitive tasks, assessing different domains, and demonstrated enhanced cognition‐related activation of motor areas both in patients with JME and their siblings. Although the episodic memory paradigm entailed a joystick response, the language task was covert, suggesting that motor hyperactivation may occur independent of cognitive domain, and in the absence of concomitant overt motor output. More widespread effects during the memory task indicate enhancement through hand motion, corroborating previous neuropsychological studies.^38^, ^39^ Given its presence in patients and siblings, motor system hyperactivation is unlikely to be a mere consequence of seizures and/or treatment, and can thus be construed as a systems‐level disease endophenotype. ROC curve analyses on quantitative metrics of motor recruitment indicated high levels of discrimination of both JME patients and their siblings from controls, providing further validation of its endophenotypic potential. From a neurobiological perspective, our findings substantiate an atypical link between cognitive processing and motor activity in JME, and suggest that its underlying determinants are genetic.

In line with the definition of endophenotype,^7^ cognition‐related motor system hyperactivity qualifies as a trait associated with JME at the population level, but is likely not sufficient to develop the disease. Furthermore, group differences in neuropsychological test scores were subtle. Thus, although enhanced motor activation in JME and siblings may specifically occur *during* the execution of cognitive tasks, it may not directly influence cognitive performance levels, or explain cognitive dysfunction per se. It is important to note, however, that we detected more marked motor activation in patients with ongoing seizures, particularly for memory encoding and the combined model across tasks. Post hoc exploratory analyses also identified a significant association between stronger motor activation and longer time since last seizure. Overall, such findings indicate trait modulation by disease activity, suggest its proximity to the final symptomatic phenotype of JME, that is, seizures with characteristic motor components (often triggered by cognitive activities), and further corroborate relevance of the identified trait to the underlying pathological mechanisms.

The study groups did not differ with respect to potential confounding factors, including task performance, joystick reaction times, frontal lobe lateralization for language and verbal encoding, and motor system LIs in all task conditions. Sensitivity analyses overall excluded a significant influence of age, sex, and handedness, as well as time of MRI acquisition. The absence of significant correlations between individual motor activation estimates and either the verbal processing LIs or the motor system LIs (a) points to independence of task‐related motor effects and verbal hemispheric dominance, and (b) suggests that enhanced motor recruitment in JME and siblings may be underpinned by localized activation surges, rather than altered interhemispheric distribution of motor resources.

Besides motor system effects, our investigation detected widespread hyperactivation of prefronto‐temporo‐parietal cortices in JME compared to controls, particularly for memory fMRI. Because these traits were not common to patients and their siblings, they do not constitute an endophenotype of JME. Previous work in frontal and temporal lobe epilepsy detected enhanced frontotemporal activation during memory encoding,^31^, ^40^ which appeared more pronounced in patients with nonimpaired memory, and was proposed as a signature of functional reorganization.^40^ By analogy, patterns of increased fronto‐temporo‐parietal activity in JME may also represent a compensatory mechanism, supporting normal function. Confirmation of these hypotheses shall be sought in future studies, which may also investigate the influence of material type (verbal vs nonverbal) on the laterality of functional reorganization. As our analysis focused on group differences *across* item categories, we could not address this aspect.

Despite the relatively homogeneous clinical presentation, the genetic basis of JME remains elusive.^1^ Mendelian JME‐associated genes have been identified, but account for only a modest proportion of patients.^41^ Currently, JME is regarded as a polygenetic disorder.^1^ Combining imaging and genetics via endophenotypes provides a novel framework to link genetic factors and disease‐associated quantifiable traits,^42^, ^43^ increasing analytical power and enhancing the success of genetic investigations. Such strategy contributed to the identification of gene polymorphisms linked to hippocampal volume in Alzheimer's disease^44^, ^45^ and altered prefrontal and striatal activation in schizophrenia,^46^ thereby improving our understanding of the neural correlates of disease susceptibility. Hence, the fMRI endophenotype identified in our study offers a suitable, measurable trait to inform future investigations into the genetic architecture of the underlying mechanisms contributing to systems‐level abnormalities in JME. It is notable that the functional imaging patterns reported here were derived from tasks assessing expressive language and episodic memory, high‐order cognitive functions often affected across epilepsies. Paradigms addressing these functions may be more widely and readily available in epilepsy centers compared to more complex working memory paradigms.^23^

Our study has limitations. Owing to ethics restrictions, EEG recordings were not available for siblings who participated in this study. Subclinical epileptiform discharges may be present in up to 20% of unaffected JME siblings^47^ in the absence of a history of seizures, suggesting that interictal discharges may be part of a wider “disease‐related spectrum.” On the other hand, previous investigations using concomitant video‐EEG during neuropsychological testing in patients with JME and their unaffected siblings^18^ did not detect an influence of interictal epileptiform discharges on cognitive performance. The aim of our investigation was not to demonstrate that findings are independent of potential subtle interictal activity, but rather to (a) identify abnormal cognitive functional imaging traits in patients with JME and (b) assess commonalities between patients and siblings, pointing to an underlying genetic etiology.

We compared JME patients with and without ongoing seizures in the year prior to the investigation, detecting more prominent motor activation in the former. Of note, ~90% of patients with poor seizure control experienced absences, in addition to myoclonus and generalized tonic‐clonic seizures (GTCSs); furthermore, almost the entirety of JME patients with history of absences were not seizure‐free, corroborating previous findings that absences are an unfavorable prognostic marker in JME.^48^ Unfortunately, however, such a phenotypic overlap in our cohort prevented us from disentangling unique absence‐related effects.

To further characterize the spectrum of motor hyperactivation in JME, additional analyses may benefit from including motor paradigms with minimal cognitive demands, such as finger tapping. Future investigations should establish whether motor system hyperactivation is specific to JME, or is common to several GGE syndromes, which may also present with praxis induction. Recent EEG‐fMRI research in a mixed GGE series, including patients with JME or epilepsy with GTCSs alone, suggests hypersynchrony of sensory‐motor networks in patients and their relatives.^49^ The latter study, however, included patients with motor seizures (myoclonus/GTCS) as primary ictal manifestation, and did not analyze GGE syndromes in which myoclonus is less common, such as absence epilepsies. In addition, resting‐state fMRI may characterize the underlying organization of functional networks at rest, but cannot examine cognitive trigger factors.

In conclusion, we demonstrate hyperactivation of the motor system during different cognitive tasks in patients with JME and their siblings, implicating trait heritability and validating the latter as a JME endophenotype. Our study offers a suitable, quantitative trait to inform future investigations into the genetic architecture of systems‐level abnormalities in JME.

## CONFLICT OF INTEREST

Dr Duncan served on the scientific advisory boards for and/or received funding for travel from GE Healthcare, GSK, Eisai, and UCB. Dr Koepp served on a scientific advisory board of GE Healthcare and has received honoraria for lectures from Eisai, Bial, Novartis, and UCB. The remaining authors have no conflict of interest. We confirm that we have read the Journal's position on issues involved in ethical publication and affirm that this report is consistent with those guidelines.

## Supporting information

Table S1

Table S2

Table S3

Table S4

Table S5

Appendix S1
