# Supplementary material for: Motor hyperactivation during cognitive tasks: An endophenotype of juvenile myoclonic epilepsy
Source: Epilepsia. 2020 Jun 25;61(7):1438–52. doi: 10.1111/epi.16575 (PMC7681252; doi:10.1111/epi.16575)
Supplement: Supplementary file 1 — Table S1 [file EPI-61-1438-s001.docx]

**Supplementary Table 1. Patient Clinical Characteristics**

| *Age* | *Sex* | *Age at onset* | *Disease duration* | *AEDs at time of investigation* |
| --- | --- | --- | --- | --- |
| 41 | .00 | 11.00 | 30.00 | VPA 800, LEV 1000, CLB prn |
| 30 | .00 | 13.00 | 17.00 | LMT 400, LEV 250 |
| 29 | .00 | 15.00 | 14.00 | VPA 2200, CLN 1mg prn |
| 49 | 1.00 | 20.00 | 29.00 | LEV 1000, LMT 100, VPA 2000 |
| 25 | 1.00 | N/A | N/A | VPA 600 |
| 25 | 1.00 | 11.00 | 14.00 | VPA 2400, LMT 300, ZNS 450 |
| 32 | .00 | 16.00 | 16.00 | VPA 200, LMT 400 |
| 36 | 1.00 | N/A | N/A | VPA 600 |
| 49 | 1.00 | 14.00 | 35.00 | LEV 2000, VPA 800 |
| 32 | 1.00 | 13.00 | 19.00 | VPA 2000, LMT 400, LEV 500 |
| 22 | 1.00 | 16.00 | 6.00 | LEV 2000, VPA 2000 |
| 32 | 1.00 | 16.00 | 16.00 | VPA 2000 |
| 29 | 1.00 | 16.00 | 13.00 | none for 1 year |
| 36 | 1.00 | 16.00 | 20.00 | VPA 2000, LMT 150 |
| 45 | .00 | 7.00 | 38.00 | LEV 1000, VPA 800 |
| 26 | .00 | 15.00 | 11.00 | VPA 400, LMT 200, ZNS 400 |
| 40 | .00 | 7.00 | 33.00 | VPA 1000 |
| 41 | .00 | 17.00 | 24.00 | PHT 300 |
| 53 | 1.00 | 8.00 | 45.00 | VPA 1000, LEV 2000, LTG 100 |
| 39 | .00 | 13.00 | 26.00 | LEV 1000, VPA 1000 |
| 27 | .00 | 16.00 | 11.00 | OXC 2100, CLB 25 |
| 64 | .00 | 20.00 | 44.00 | LEV 2000 |
| 25 | .00 | 15.00 | 10.00 | LMT 200 |
| 25 | 1.00 | 16.00 | 9.00 | VPA 2000, LEV 1500 |
| 30 | 1.00 | 18.00 | 12.00 | VPA 1800 |
| 54 | .00 | 15.00 | 39.00 | VPA 500 |
| 36 | 1.00 | 7.00 | 29.00 | LTG 600, LEV 3500 |
| 22 | .00 | 9.00 | 13.00 | VPA 1000, DZP 5 |
| 22 | .00 | 14.00 | 8.00 | LEV 1500, LMT 600 |
| 35 | 1.00 | 19.00 | 16.00 | VPA 1000 |
| 39 | .00 | 12.00 | 27.00 | VPA 400, LMT 450 |
| 24 | .00 | 15.00 | 9.00 | OXC 300, ZNS 250, LEV 4000, LMT 200 |

Abbreviations: CLB= clobazam; CLN= clonazepam; DZP= diazepam; LEV= levetiracetam; LMT= lamotrigine; OXC= oxcarbazepine; PHT= phenytoin; prn *pro re nata,* i.e. as needed; VPA= sodium valproate; ZNS= zonisamide.
