# Supplementary material for: Motor hyperactivation during cognitive tasks: An endophenotype of juvenile myoclonic epilepsy
Source: Epilepsia. 2020 Jun 25;61(7):1438–52. doi: 10.1111/epi.16575 (PMC7681252; doi:10.1111/epi.16575)
Supplement: Supplementary file 2 — Table S2 [file EPI-61-1438-s002.docx]

**Supplementary Table 2. Group comparisons for memory activations: fMRI coordinates and statistics**

| *Region* | MNI coordinates  (x y z) | *Z*-score | *P* value | MNI coordinates  (x y z) | *Z*-score | *P* value |
| --- | --- | --- | --- | --- | --- | --- |
|  | ***Left hemisphere*** | | | ***Right hemisphere*** | | |
| Main effect of group *(F contrast)* |  |  |  |  |  |  |
| *Precentral gyrus* | -30 13 70 | 3.66  (3.58) | **0.001**  (**0.002**) | 57 2 4 | 3.45  (3.54) | **0.003**  (**0.002**) |
|  | -36 -16 46 | 3.32  (3.25) | **0.004**  (**0.005**) | 12 2 61 | 2.88  (2.76) | **0.015**  (**0.021**) |
|  | -45 -13 58 | 3.20  (3.17) | **0.006**  (**0.007**) | 51 -4 43 | 2.87  (2.79) | **0.015**  (**0.019**) |
|  | -54 10 46 | 3.15  (3.13) | **0.008**  (**0.007**) | 15 20 52 | 2.71  (2.65) | **0.024**  (**0.027**) |
| *Supplementary motor area* | -6 -1 64 | 3.46  (3.49) | **0.003**  (**0.002**) | 3 2 55 | 3.18  (3.15) | **0.005**  (**0.007**) |
|  | -9 8 52 | 3.46  (3.42) | **0.003**  (**0.003**) | 3 -1 70 | 3.17  (3.02) | **0.006**  (**0.010**) |
|  | -1 2 55 | 3.18  (3.09) | **0.006**  (**0.008**) |  |  |  |
|  | -12 31 64 | 2.76  (2.85) | **0.020**  (**0.016**) |  |  |  |
|  |  |  |  |  |  |  |
| *Middle frontal gyrus* | -39 44 34 | 3.08  (3.30) | 0.001^  (<0.001) |  |  |  |
|  | -30 8 64 | 2.83  (2.99) | 0.002^  (0.001)^ |  |  |  |
| *Inferior frontal gyrus* | -51 8 -5 | 3.05  (3.12) | 0.001  (0.001) |  |  |  |
| *Middle cingulate gyrus* | -1 14 34 | 3.43  (3.32) | <0.001  (<0.001) |  |  |  |
| *Rolandic operculum* |  |  |  | 51 -25 16 | 3.52  (3.58) | <0.001  (<0.001) |
| *Supramarginal gyrus* | -54 -34 58 | 3.03  (2.81) | 0.001^  (0.003)^ |  |  |  |
| *Angular gyrus* | -54 -55 52 | 2.84  (2.72) | 0.002^  (0.003)^ |  |  |  |
| *Precuneus* | -6 -52 58 | 3.48  (3.65) | <0.001  (<0.001) |  |  |  |
| *Superior temporal gyrus* | -63 -22 10 | 3.24  (3.40) | 0.001  (<0.001) | 63 -19 13 | 3.81  (3.82) | <0.001  (<0.001) |
| *Insula* |  |  |  | 39 11 4 | 2.88  (2.87) | 0.002^  (0.002)^ |
| *Lingual gyrus* | -3 -79 -2 | 3.08  (3.40) | 0.001  (<0.001) | 18 -67 1 | 3.17  (3.46) | 0.001  (<0.001) |
| *Cerebellum* |  |  |  | 6 -82 -23 | 2.72  (2.99) | 0.003^  (0.001)^ |
| JME > CTR |  |  |  |  |  |  |
| *Precentral gyrus* | -45 -13 58 | 3.63  (3.43) | **0.001**  **(0.003)** | 57 2 4 | 3.98  (3.43) | **<0.001**  **(0.003)** |
|  | -48 5 -2 | 3.57  (3.51) | **0.002**  **(0.002)** | 51 -4 40 | 3.42  (3.33) | **0.003**  (**0.004**) |
|  | -27 -16 70 | 3.50  (3.38) | **0.002**  **(0.003)** | 54 2 19 | 3.06  (3.30) | **0.008**  (**0.004)** |
|  | -9 -31 70 | 3.44  (3.42) | **0.002**  **(0.003)** | 24 -16 67 | 2.92  (2.86) | **0.012**  **(0.014)** |
| *Supplementary motor area* | -6 -4 64 | 3.81  (3.57) | **0.001**  **(0.002)** | 3 -1 70 | 3.59  (3.44) | **0.002**  **(0.002)** |
|  | -9 -10 76 | 3.38  (3.36) | **0.003**  **(0.003)** | 6 11 52 | 3.31  (3.05) | **0.004**  **(0.008)** |
|  | -12 -7 43 | 2.70  (2.65) | **0.020**  **(0.023)** |  |  |  |
|  |  |  |  |  |  |  |
| *Superior frontal gyrus* | -15 50 49 | 3.36  (3.37) | <0.001^  (<0.001)^ | 24 47 46 | 2.91  (2.93) | 0.002  (0.002) |
| *Middle frontal gyrus* | -42 44 34 | 3.63  (3.77) | <0.001  (<0.001) | 39 41 28 | 3.15  (3.15) | 0.001  (0.001) |
| *Inferior frontal gyrus* | -51 8 -5 | 3.54  (3.51) | <0.001  (<0.001) | 51 20 25 | 2.89  (2.95) | 0.002^  (0.002)^ |
| *Postcentral gyrus* | -18 -31 76 | 3.06  (3.08) | 0.001  (0.001) | 15 -34 79 | 3.22  (3.40) | 0.001^  (<0.001)^ |
| *Superior parietal lobule* | -45 -49 64 | 3.01  (2.89) | 0.001  (0.002) | 27 -43 55 | 3.31  (3.39) | <0.001  (<0.001) |
| *Supramarginal gyrus* | -54 -37 58 | 3.43  (3.19) | <0.001  (0.001) |  |  |  |
| *Superior temporal gyrus* | -63 -22 10 | 4.11  (4.21) | <0.001  (<0.001) | 63 -19 13 | 4.21  (4.22) | <0.001  (<0.001) |
| *Heschl’s gyrus* | -42 -19 7 | 3.13  (3.16) | 0.001  (0.001) |  |  |  |
| *Rolandic operculum* | -66 -31 34 | 3.00  (3.03) | 0.001  (0.001) | 51 -25 16 | 3.78  (3.74) | <0.001  (<0.001) |
| *Anterior thalamus* |  |  |  | 12 -1 10 | 2.78  (2.77) | 0.003^  (0.003)^ |
| JME non-seizure free > JME seizure free |  |  |  |  |  |  |
| *Precentral gyrus* | -60 -4 40 | 2.86  (2.86) | **0.015**  (**0.015**) | 36 -10 43 | 3.28  (3.17) | **0.004**  **(0.006)** |
|  | -15 -13 67 | 2.60  (2.78) | **0.029**  (**0.018**) | 33 -19 46 | 3.26  (3.01) | **0.005**  (**0.010**) |
| *Supplementary motor area* |  |  |  | 12 -25 64 | 2.57  (2.41) | **0.031**  (**0.044**) |
|  |  |  |  |  |  |  |
| *Superior frontal gyrus* | -12 8 61 | 2.81  (2.85) | 0.003^  (0.002)^ |  |  |  |
| *Middle frontal gyrus* | -33 41 46 | 2.84  (2.71) | 0.002^  (0.003)^ |  |  |  |
| *Superior parietal lobule* |  |  |  | 30 -43 52 | 3.13  (3.05) | 0.001  (0.001)^ |
| *Middle cingulate gyrus* |  |  |  | 12 17 22 | 3.13  (2.78) | 0.001  (0.003)^ |
| *Posterior cingulate cortex* | -12 -49 25 | 3.28  (3.16) | 0.001  (0.001) | 9 -37 37 | 3.02  (2.81) | 0.001  (0.002) |
| JME non-seizure free > CTR |  |  |  |  |  |  |
| *Precentral gyrus* | -57 -4 37 | 3.31  (3.33) | **0.004**  (**0.004**) | 39 -10 46 | 3.39  (3.39) | **0.003**  (**0.004**) |
|  | -15 -13 67 | 3.23  (3.33) | **0.005**  (**0.004**) | 54 -1 34 | 3.20  (3.14) | **0.007**  (**0.007**) |
|  | -27 -16 70 | 2.98  (2.99) | **0.011**  (**0.010**) | 21 -16 70 | 2.77  (2.84) | **0.019**  (**0.015**) |
|  | -45 -10 58 | 2.92  (2.95) | **0.012**  (**0.011**) | 27 -19 58 | 2.75  (2.78) | **0.020**  (**0.018**) |
| *Supplementary motor area* | -12 2 58 | 3.54  (3.57) | **0.002**  (**0.002**) | 3 2 70 | 3.52  (3.56) | **0.002**  (**0.002**) |
|  | -1 -1 70 | 3.32  (3.35) | **0.004**  (**0.004**) | 12 17 58 | 3.32  (3.31) | **0.004**  (**0.004**) |
|  | -3 -10 46 | 2.85  (2.83) | **0.015**  (**0.016**) | 9 -10 70 | 3.14  (3.20) | **0.001**  (**0.006**) |
|  |  |  |  |  |  |  |
| *Superior frontal gyrus* | -15 50 49 | 3.06  (3.02) | 0.001  (0.001)^ | 6 32 34 | 2.95  (2.91) | 0.002  (0.002) |
| *Middle frontal gyrus* | -42 44 34 | 3.82  (3.84) | <0.001  (<0.001) | 33 41 22 | 3.01  (2.99) | 0.001  (0.001) |
| *Inferior frontal gyrus* | -45 5 -5 | 2.79  (2.81) | 0.003^  (0.003)^ | 51 17 25 | 3.27  (3.24) | 0.001  (0.001) |
| *Middle cingulate cortex* |  |  |  | 18 23 25 | 3.15  (3.18) | 0.001  (0.001) |
| *Superior parietal lobule* |  |  |  | 30 -43 52 | 3.83  (3.82) | <0.001  (<0.001) |
| *Supramarginal gyrus* | -60 -34 37 | 2.86  (2.87) | 0.002  (0.002) | 48 -31 34 | 3.03  (3.02) | 0.001  (0.001)^ |
| *Superior temporal gyrus* | -63 -22 10 | 3.08  (3.16) | 0.001^^^  (0.001) | 63 -19 10 | 3.45  (3.44) | <0.001  (<0.001) |
| *Insula* |  |  |  | 36 11 4 | 2.87  (2.89) | 0.002  (0.002) |
| JME seizure free > CTR |  |  |  |  |  |  |
| *Precentral gyrus* | -48 5 1 | 3.13  (2.94) | **0.007**  (**0.012**) | 57 2 4 | 3.85  (3.63) | **0.001**  **(0.001)** |
|  | -42 -16 55 | 3.13  (2.78) | **0.007**  (**0.018**) |  |  |  |
|  | -33 -25 73 | 2.71  (2.57) | **0.022**  (**0.031**) |  |  |  |
|  | -15 -31 64 | 2.60  (2.54) | **0.028**  (**0.032**) |  |  |  |
| *Supplementary motor area* | -3 -4 64 | 2.78  (2.30) | **0.018**  **(0.057)** |  |  |  |
|  |  |  |  |  |  |  |
| *Inferior frontal gyrus* | -39 32 -2 | 2.81  (3.12) | 0.002^  (0.001) |  |  |  |
| *Postcentral gyrus* | -15 -31 79 | 3.00  (3.18) | 0.001  (0.001) | 54 -22 16 | 3.31  (3.19) | <0.001  (0.001) |
|  |  |  |  | 15 -34 79 | 3.53  (3.75) | <0.001^  (<0.001) |
| *Supramarginal gyrus* | -54 -40 58 | 4.06  (3.63) | <0.001  (<0.001) |  |  |  |
| *Superior parietal lobule* |  |  |  | 30 -40 73 | 2.81  (3.08) | 0.002^  (0.001) |
| *Superior temporal gyrus* | -60 -22 10 | 3.55  (3.66) | <0.001  (<0.001) |  |  |  |
| *Middle temporal gyrus* | -57 -70 1 | 3.41  (3.56) | <0.001^  (<0.001)^ |  |  |  |
| SIB > CTR |  |  |  |  |  |  |
| *Precentral gyrus* | -30 -13 70 | 3.63  (3.38) | **0.002**  (**0.004**) |  |  |  |
|  | -42 -16 64 | 2.52  (3.18) | **0.036**  (**0.006**) |  |  |  |
|  |  |  |  |  |  |  |
| *Inferior frontal gyrus*  *(bordering precentral gyrus)* |  |  |  | 33 32 8 | 2.82  (2.61) | 0.002^  (0.004)^ |
| *Cerebellum* |  |  |  | 9 -82 -23 | 3.01  (3.01) | 0.001^  (0.001)^ |
| SIB < CTR |  |  |  |  |  |  |
| *Lingual gyrus* |  |  |  | 21 -64 2 | 3.47  (3.81) | <0.001  (<0.001) |
| Conjunction  (JME & SIB > CTR) |  |  |  |  |  |  |
| *Precentral gyrus* | 27 -16 70 | 3.33  (3.29) | **0.003**  (**0.004**) |  |  |  |
|  | -42 -19 61 | 2.72  (3.13) | **0.019**  (**0.006**) |  |  |  |
|  |  |  |  |  |  |  |
| *Postcentral gyrus* | -54 -34 55 | 2.78  (2.77) | 0.002***^***  (0.003)***^*** |  |  |  |
| *Superior temporal gyrus* |  |  |  | 66 -22 7 | 3.01  (2.77) | 0.001***^***  (0.003)***^*** |

Abbreviations: CTR= controls; JME= patients with juvenile myoclonic epilepsy; MNI= Montreal Neurological Institute; SIB= siblings of patients with juvenile myoclonic epilepsy. Coordinates of fMRI activation differences are provided in MNI space. *P*-values for differences in motor system activation (precentral gyrus, supplementary motor area), all reported in bold font, are family-wise error rate (FWE) corrected for multiple comparisons within small volume, using a 12-mm diameter sphere centred on local maxima. *P*-values not in bold, pertaining to activation differences for non-motor areas, are reported as uncorrected for multiple comparisons (*p*<0.005, k=20; if ^: peak *p<*0.005, but related cluster <20 voxels). Z-scores and *P-*values in brackets refer to repeat group analyses including age, sex and handedness as regressors of no interest, which produced virtually identical results. There were no areas of increased activation in controls compared to patients with JME and JME subgroups; similarly, there were no areas of increased activation in seizure-free JME patients compared to those with ongoing seizures.
