# Supplementary material for: Motor hyperactivation during cognitive tasks: An endophenotype of juvenile myoclonic epilepsy
Source: Epilepsia. 2020 Jun 25;61(7):1438–52. doi: 10.1111/epi.16575 (PMC7681252; doi:10.1111/epi.16575)
Supplement: Supplementary file 3 — Table S3 [file EPI-61-1438-s003.docx]

**Supplementary Table 3. Group comparisons for language activations: fMRI coordinates and statistics**

| *Region* | MNI coordinates  (x y z) | *Z*-score | *P* value | MNI coordinates  (x y z) | *Z*-score | *P* value |
| --- | --- | --- | --- | --- | --- | --- |
|  | ***Left hemisphere*** | | | ***Right hemisphere*** | | |
| Main effect of group *(F contrast)* |  |  |  |  |  |  |
| *Supplementary motor area* | -12 2 55 | 3.23  (2.80) | **0.006**  **(0.021)** |  |  |  |
| *Precentral gyrus* | -45 -1 52 | 2.93  (2.55) | **0.015**  **(0.038)** |  |  |  |
| JME > CTR |  |  |  |  |  |  |
| *Supplementary motor area* | -9 2 55 | 3.51  (3.17) | **0.002**  **(0.007)** |  |  |  |
|  | -9 -4 67 | 3.02  (2.74) | **0.010**  **(0.021)** |  |  |  |
| *Precentral gyrus* | -60 2 31 | 2.86  (3.02) | **0.016**  **(0.010)** |  |  |  |
| JME non-seizure free > JME seizure free |  |  |  |  |  |  |
| *Precentral gyrus* | -39 -10 67 | 2.67  (3.06) | **0.025**  **(0.009)** | 27 -16 73 | 2.85  (2.87) | **0.016**  **(0.015)** |
|  |  |  |  |  |  |  |
| *Middle frontal gyrus* | -42 35 43 | 3.05  (3.01) | 0.001^  (0.001)^ |  |  |  |
| JME non-seizure free > CTR |  |  |  |  |  |  |
| *Supplementary motor area* | -12 -4 67 | 2.73  (2.82) | **0.026**  **(0.017)** |  |  |  |
| *Precentral gyrus* | -36 -10 67 | 2.85  (2.93) | **0.016**  **(0.013)** |  |  |  |
|  |  |  |  |  |  |  |
| *Middle frontal gyrus* | -39 38 37 | 2.93  (2.95) | 0.002^  (0.002) |  |  |  |
| JME seizure free > CTR |  |  |  |  |  |  |
| *Supplementary motor area* | -9 2 55 | 4.41  (3.61) | **<0.001**  **(0.002)** |  |  |  |
| *Precentral gyrus* | -60 2 34 | 2.93  (3.16) | **0.013**  **(0.007)** |  |  |  |
| SIB > CTR |  |  |  |  |  |  |
| *Supplementary motor area* | -6 -1 70 | 2.82  (2.69) | **0.018**  **(0.025)** |  |  |  |
| *Precentral gyrus* | -45 -1 52 | 3.32  (2.94) | **0.004**  **(0.013)** |  |  |  |
|  | -60 8 28 | 2.79  (2.48) | **0.019**  **(0.041)** |  |  |  |
|  |  |  |  |  |  |  |
| *Middle frontal gyrus* | -42 47 28 | 3.14  (3.07) | 0.001^  (0.001)^ |  |  |  |
| *Temporal pole* | -45 8 -35 | 2.99  (2.88) | 0.001^  (0.002)^ |  |  |  |
| Conjunction  (JME & SIB > CTR) |  |  |  |  |  |  |
| *Supplementary motor area* | -9 -1 70 | 2.51  (2.35) | **0.036**  **(0.050)** |  |  |  |
| *Precentral gyrus* | -45 -7 49 | 2.47  (2.33) | **0.040**  **(0.051)** |  |  |  |

Abbreviations: CTR= controls; JME= patients with juvenile myoclonic epilepsy; MNI= Montreal Neurological Institute; SIB= siblings of patients with juvenile myoclonic epilepsy. Coordinates of fMRI activation differences are provided in MNI space. *P*-values for differences in motor system activation (precentral gyrus, supplementary motor area), all reported in bold font, are family-wise error rate (FWE) corrected for multiple comparisons within small volume, using a 12-mm diameter sphere centred on local maxima. *P*-values not in bold, pertaining to activation differences for non-motor areas, are reported as uncorrected for multiple comparisons (*p*<0.005, k=20; if ^: peak *p<*0.005, but related cluster <20 voxels). Z-scores and *P-*values in brackets refer to repeat group analyses including age, sex and handedness as regressors of no interest, which produced virtually identical results. There were no areas of increased activation in controls compared to JME patients, JME subgroups and JME siblings; similarly, there were no areas of increased activation in seizure-free JME patients compared to those with ongoing seizures.
