# Supplementary material for: Motor hyperactivation during cognitive tasks: An endophenotype of juvenile myoclonic epilepsy
Source: Epilepsia. 2020 Jun 25;61(7):1438–52. doi: 10.1111/epi.16575 (PMC7681252; doi:10.1111/epi.16575)
Supplement: Supplementary file 4 — Table S4 [file EPI-61-1438-s004.docx]

**Supplementary Table 4. Group comparisons for the combined activation model across tasks: fMRI coordinates and statistics**

| *Region* | MNI coordinates  (x y z) | *Z*-score | | *P* value | MNI coordinates  (x y z) | *Z*-score | *P* value |
| --- | --- | --- | --- | --- | --- | --- | --- |
|  | ***Left hemisphere*** | | | | ***Right hemisphere*** | | |
| Main effect of group *(F contrast)* |  |  |  | |  |  |  |
| *Supplementary motor area* | -9 -4 64 | 3.55  (3.52) | | **0.002**  (**0.002**) | 3 2 55 | 3.24  (2.57) | **0.006**  (**0.031**) |
|  | -9 8 52 | 3.35  (3.32) | | **0.004**  (**0.005**) | 3 -4 70 | 2.64  (2.53) | **0.004**  (**0.034)** |
| *Precentral gyrus* | -30 -13 70 | 3.66  (3.68) | | **0.001**  (**0.001**) | 15 5 67 | 2.69  (2.49) | **0.028**  (**0.037**) |
|  | -45 -13 58 | 3.07  (3.05) | | **0.010**  (**0.010**) | 12 -7 73 | 2.67  (2.44) | **0.029**  (**0.041**) |
|  | -57 5 4 | 2.71  (2.68) | | **0.026**  (**0.028**) |  |  |  |
|  |  |  | |  |  |  |  |
| *Middle frontal gyrus* | -39 41 31 | 3.28  (3.26) | | 0.001  (0.001) |  |  |  |
| *Inferior frontal gyrus* | -45 5 -5 | 2.86  (2.83) | | 0.002^  (0.002)^ |  |  |  |
| *Postcentral gyrus* | -66 -31 34 | 2.71  (2.68) | | 0.003^  (0.004)^ |  |  |  |
| *Posterior paracentral lobule* | -18 -34 79 | 2.71  (2.68) | | 0.003^  (0.004)^ |  |  |  |
| *Rolandic operculum* |  |  | |  | 51 -25 19 | 2.71  (2.68) | 0.003^  (0.004)^ |
| *Precuneus* | -3 -52 58 | 2.97  (2.96) | | 0.001^  (0.002)^ |  |  |  |
| *Superior temporal gyrus* | -63 -22 10 | 3.03  (3.03) | | 0.001^  (0.001)^ | 51 17 -14 | 3.42  (3.37) | <0.001  (<0.001) |
| *Insula* |  |  | |  | 36 11 4 | 3.07  (3.06) | 0.001  (0.001) |
| JME > CTR |  |  | |  |  |  |  |
| *Supplementary motor area* | -3 2 55 | 4.03  (3.73) | | **<0.001**  (**0.001**) | 3 2 55 | 3.79  (3.52) | **0.001**  (**0.002**) |
|  | -9 -4 64 | 4.00  (3.77) | | **<0.001**  (**0.001**) | 3 -7 67 | 3.27  (3.12) | **0.004**  (**0.007**) |
| *Precentral gyrus* | -45 -13 58 | 3.58  (3.45) | | **0.002**  (**0.003**) | 12 -10 73 | 3.30  (3.23) | **0.004**  (**0.005**) |
|  | -27 -16 70 | 3.50  (3.34) | | **0.002**  (**0.004**) | 15 2 70 | 3.13  (2.92) | **0.007**  (**0.012**) |
|  | -42 -7 46 | 3.37  (3.23) | | **0.003**  (**0.005**) | 54 2 37 | 3.07  (2.98) | **0.008**  (**0.010**) |
|  | -57 5 4 | 3.29  (3.21) | | **0.004**  (**0.005**) | 51 5 52 | 2.90  (2.65) | **0.013**  (**0.025**) |
|  |  |  | |  |  |  |  |
| *Superior frontal gyrus* | -15 50 49 | 3.31  (3.21) | | <0.001  (0.001) | 12 29 37 | 3.09  (3.14) | 0.001  (0.001) |
|  |  |  | |  | 24 47 46 | 2.90  (2.94) | 0.002  (0.002) |
| *Middle frontal gyrus* | -39 41 31 | 3.87  (3.85) | | <0.001  (<0.001) | 39 44 28 | 3.14  (3.10) | 0.001  (0.001) |
| *Inferior frontal gyrus* | -45 5 -5 | 3.44  (3.49) | | <0.001  (<0.001) |  |  |  |
| *Postcentral gyrus* | -66 -31 34 | 3.05  (2.99) | | 0.001  (0.001) |  |  |  |
| *Posterior paracentral lobule* | -18 -31 79 | 3.24  (3.20) | | 0.001  (0.001) |  |  |  |
| *Rolandic operculum* |  |  | |  | 54 2 4 | 3.33  (3.40) | <0.001  (<0.001) |
| *Superior parietal lobule* | -27 -52 58 | 2.90  (2.94) | | 0.002^  (0.002)^ |  |  |  |
| *Supramarginal gyrus* | -54 -34 49 | 3.17  (2.99) | | 0.001  (0.001) |  |  |  |
| *Middle cingulate gyrus* | -3 8 31 | 3.04  (3.04) | | 0.001  (0.001) |  |  |  |
| *Superior temporal gyrus* | -63 -22 10 | 3.70  (3.70) | | <0.001  (<0.001) | 51 17 -14 | 3.82  (3.76) | <0.001  (<0.001) |
| *Insula* |  |  | |  | 36 11 4 | 3.46  (3.58) | <0.001  (<0.001) |
|  |  |  | |  | 63 -19 16 | 3.05  (3.19) | 0.001  (0.001) |
| JME non-seizure free > JME seizure free |  |  | |  |  |  |  |
| *Supplementary motor area* | -12 -13 67 | 2.65  (2.87) | | **0.027**  (**0.016**) | 12 2 46 | 2.70  (2.64) | **0.024**  (**0.028**) |
| *Precentral gyrus* | -33 -10 61 | 2.86  (3.38) | | **0.016**  (**0.004**) | 36 -10 43 | 2.81  (2.80) | **0.018**  (**0.018**) |
|  | -51 -7 40 | 2.52  (2.69) | | **0.037**  (**0.025**) |  |  |  |
|  |  |  | |  |  |  |  |
| *Superior frontal gyrus* | -9 59 37 | 2.81  (2.66) | | 0.002^  (0.004)^ | 21 23 61 | 2.85  (2.66) | 0.002  (0.004)^ |
| *Middle frontal gyrus* | -39 35 40 | 3.25  (3.11) | | 0.001  (0.001) | 36 20 37 | 2.96  (2.68) | 0.002^  (0.004)^ |
| *Inferior frontal gyrus* |  |  | |  | 48 17 25 | 3.31  (3.02) | <0.001  (0.001) |
| *Middle cingulate gyrus* | -15 -10 37 | 2.84  (2.71) | | 0.002  (0.003)^ | 12 17 22 | 3.10  (2.72) | 0.001  (0.003)^ |
| *Posterior cingulate gyrus* | -12 -49 25 | 3.60  (3.42) | | <0.001  (<0.001) |  |  |  |
|  | -9 -34 37 | 3.31  (2.91) | | <0.001  (0.002) | 9 -34 37 | 3.44  (3.01) | <0.001  (0.001) |
| *Supramarginal gyrus* | -60 -31 34 | 2.78  (2.90) | | 0.003^  (0.002) |  |  |  |
| JME non-seizure free > CTR |  |  | |  |  |  |  |
| *Supplementary motor area* | -12 -1 58 | 3.88  (4.07) | | **0.001**  **(<0.001**) | 12 17 58 | 3.43  (3.40) | **0.** **003**  (**0.003**) |
|  | -9 -10 67 | 3.79  (3.87) | | **0.001**  (**0.001**) | 12 -10 70 | 3.28  (3.36) | **0.** **005**  (**0.004**) |
|  | -15 -13 67 | 3.21  (3.37) | | **0.006**  (**0.004**) | 3 -7 67 | 3.16  (3.18) | **0.007**  (**0.006**) |
| *Precentral gyrus* | -33 -13 67 | 3.42  (3.43) | | **0.003**  (**0.003**) | 54 -1 31 | 3.13  (3.02) | **0.008**  (**0.010**) |
|  | -42 -7 46 | 3.37  (3.37) | | **0.004**  (**0.004**) | 39 -10 43 | 3.04  (2.99) | **0.010**  (**0.011**) |
|  | -45 13 58 | 3.25  (3.20) | | **0.005**  (**0.006**) | 21 -16 76 | 2.73  (2.66) | **0.022**  (**0.027**) |
|  | -21 -16 73 | 3.07  (3.10) | | **0.009**  (**0.008**) |  |  |  |
|  |  |  | |  |  |  |  |
| *Middle frontal gyrus* | -42 41 34 | 4.10  (4.05) | | <0.001  (<0.001) | 33 41 22 | 3.11  (3.09) | 0.001  (0.001) |
| *Inferior frontal gyrus* | -45 5 -5 | 2.99  (2.91) | | 0.001  (0.002)^ | 48 17 25 | 3.58  (3.48) | <0.001  (<0.001) |
| *Middle cingulate gyrus* |  |  | |  | 18 23 25 | 3.01  (2.96) | 0.001  (0.002) |
| *Medial orbital gyrus* | -15 20 -17 | 3.38  (3.28) | | <0.001  (<0.001) | 12 20 -17 | 3.03  (2.96) | 0.001  (0.002) |
| *Superior parietal lobule* | -24 -52 55 | 3.17  (3.10) | | 0.001^  (0.001)^ | 27 -40 52 | 3.12  (3.07) | 0.001  (0.001) |
| *Supramarginal gyrus* | -60 34 37 | 3.32  (3.33) | | <0.001  (<0.001) |  |  |  |
| *Superior temporal gyrus* | -63 -22 10 | 3.11  (3.06) | | 0.001^  (0.001)^ | 51 17 -14 | 3.10  (3.00) | 0.001  (0.001)^ |
| *Insula* | -39 8 -11 | 2.86  (2.74) | | 0.002  (0.003)^ | 33 11 4 | 3.05  (2.94) | 0.001  (0.002) |
| *Posterior thalamus* | -6 -28 2 | 3.01  (2.91) | | 0.001  (0.002)^ |  |  |  |
| *Putamen* |  |  | |  | 12 5 -5 | 2.76  (2.67) | 0.003^  (0.004)^ |
| JME seizure free > CTR |  |  | |  |  |  |  |
| *Supplementary motor area* | -3 2 55 | 3.42  (2.80) | | **0.003**  (**0.019**) | 3 2 55 | 3.07  (2.50) | **0.009**  **(0.039)** |
| *Precentral gyrus* | -48 -5 1 | 2.68  (2.78) | | **0.025**  (**0.020**) | 54 2 4 | 3.02  (3.16) | **0.010**  (**0.007**) |
|  | -57 2 4 | 2.63  (2.47) | | **0.029**  (**0.041**) |  |  |  |
|  |  |  | |  |  |  |  |
| *Inferior frontal gyrus* | -39 32 -2 | 2.85  (2.98) | | 0.002^  (0.001)^ |  |  |  |
| *Supramarginal gyrus* | -54 -40 58 | 3.06  (2.50) | | 0.001^  (*>0.005*) |  |  |  |
| *Superior temporal gyrus* | -60 -25 10 | 2.96  (2.90) | | 0.002^  (0.002)^ | 51 11 -8 | 3.19  (3.08) | 0.001  (0.001) |
| *Middle temporal gyrus* | -57 -70 1 | 2.90  (2.79) | | 0.002^  (0.003)^ |  |  |  |
| SIB > CTR |  |  | |  |  |  |  |
| *Precentral gyrus* | -30 -13 70 | 3.40  (3.16) | | **0.003**  (**0.007**) |  |  |  |
|  | -45 -4 49 | 2.75  (2.47) | | **0.021**  (**0.041**) |  |  |  |
|  | -45 -16 61 | 2.53  (2.87) | | **0.036**  **(0.002)** |  |  |  |
|  |  |  | |  |  |  |  |
| *Cerebellum* |  |  | |  | 9 82 -83 | 3.02  (3.04) | 0.001^  (0.001)^ |
| SIB < CTR |  |  | |  |  |  |  |
| *Superior parietal lobule* | -3 -52 61 | 3.06  (3.21) | | 0.001  (0.001) |  |  |  |
| Conjunction  (JME & SIB > CTR) |  |  | |  |  |  |  |
| *Precentral gyrus* | -30 -13 70 | 3.37  (3.34) | | **0.003**  (**0.004**) |  |  |  |
|  | -45 -4 49 | 2.79  (2.77) | | **0.018**  **(0.019)** |  |  |  |

Abbreviations: CTR= controls; JME= patients with juvenile myoclonic epilepsy; MNI= Montreal Neurological Institute; SIB= siblings of patients with juvenile myoclonic epilepsy. Coordinates of fMRI activation differences are provided in MNI space. *P*-values for differences in motor system activation (precentral gyrus, supplementary motor area), all reported in bold font, are family-wise error rate (FWE) corrected for multiple comparisons within small volume, using a 12-mm diameter sphere centred on local maxima. *P*-values not in bold, pertaining to activation differences for non-motor areas, are reported as uncorrected for multiple comparisons (*p*<0.005, k=20; if ^: peak *p<*0.005, but related cluster <20 voxels). Z-scores and *P-*values in brackets refer to repeat group analyses including age, sex and handedness as regressors of no interest, which produced virtually identical results. There were no areas of increased activation in controls compared to patients with JME and JME subgroups; similarly, there were no areas of increased activation in seizure-free JME patients compared to those with ongoing seizures.
