# Supplementary material for: Motor hyperactivation during cognitive tasks: An endophenotype of juvenile myoclonic epilepsy
Source: Epilepsia. 2020 Jun 25;61(7):1438–52. doi: 10.1111/epi.16575 (PMC7681252; doi:10.1111/epi.16575)
Supplement: Supplementary file 5 — Table S5 [file EPI-61-1438-s005.docx]

**Supplementary Table 5.**

**Repeat comparisons controlling for time of day of MRI acquisition: fMRI coordinates and statistics for motor effects, juxtaposed with main analysis data**

| *Region* | MNI coordinates  (x y z) | *Z*-score | *P* value | MNI coordinates  (x y z) | *Z*-score | | *P* value |
| --- | --- | --- | --- | --- | --- | --- | --- |
|  | ***Left hemisphere*** | | | ***Right hemisphere*** | | | |
| SIB > CTR, *Memory* |  |  |  |  |  |  | |
| *Precentral gyrus* | -30 -13 70 | 3.63 \| 2.96  (3.38 \| 2.86) | **0.002 \| 0.012**  (**0.004 \| 0.016**) |  |  |  | |
|  | -42 -16 64 | 2.52 \| 2.12  (3.18 \| 2.86) | **0.036 *\|* 0.083**  (**0.006 \| 0.016**) |  |  |  | |
| Conjunction (JME & SIB > CTR), *Memory* |  |  |  |  |  |  | |
| *Precentral gyrus* | 27 -16 70 | 3.33 \| 3.19  (3.29 \| 3.11) | **0.003 \| 0.005**  (**0.004 \| 0.007**) |  |  |  | |
|  | -42 -19 61 | 2.72 \| 2.32  (3.13 \| 2.80) | **0.019 \| 0.049**  (**0.006 \| 0.016**) |  |  |  | |
| SIB > CTR, *Language* |  |  |  |  |  |  | |
| *Supplementary motor area* | -6 -1 70 | 2.82 \| 2.82  (2.69 \| 2.58) | **0.018 \| 0.018**  **(0.025 \| 0.033)** |  |  |  | |
| *Precentral gyrus* | -45 -1 52 | 3.32 \| 3.30  (2.94 \| 3.01) | **0.004 \| 0.005**  **(0.013 \| 0.011)** |  |  |  | |
|  | -60 8 28 | 2.79 \| 2.64  (2.48 \| 2.39) | **0.019 \| 0.028**  **(0.041 \| 0.050)** |  |  |  | |
| Conjunction (JME & SIB > CTR), *Language* |  |  |  |  |  |  | |
| *Supplementary motor area* | -9 -1 70 | 2.51 \| 2.47  (2.35 \| 2.32) | **0.036\| 0.038**  **(0.050 \| 0.053)** |  |  |  | |
| *Precentral gyrus* | -45 -7 49 | 2.47 \| 2.37  (2.33 \| 2.24) | **0.040 \| 0.048**  **(0.051 \| 0.062)** |  |  |  | |
| SIB > CTR, *Combined model* |  |  |  |  |  |  | |
| *Precentral gyrus* | -30 -13 70 | 3.40 \| 3.39  (3.16 \| 3.16) | **0.003\| 0.003**  (**0.007 \| 0.007**) |  |  |  | |
|  | -45 -4 49 | 2.75 \| 2.71  (2.47 \| 2.44) | **0.021 \| 0.023**  (**0.041 \| 0.044**) |  |  |  | |
|  | -45 -16 61 | 2.53 \| 2.52  (2.95 \| 2.92) | **0.036 \| 0.038**  **(0.012 \| 0.014)** |  |  |  | |
| Conjunction (JME & SIB > CTR), *Combined model* |  |  |  |  |  |  | |
| *Precentral gyrus* | -30 -13 70 | 3.37 \| 3.34  (3.34 \| 3.32) | **0.003 \| 0.005**  (**0.004 \| 0.004**) |  |  |  | |
|  | -45 -4 49 | 2.79 \| 2.73  (2.77 \| 2.73) | **0.018 \| 0.021**  **(0.019 \| 0.021)** |  |  |  | |

Abbreviations: CTR= controls; MNI= Montreal Neurological Institute; SIB= siblings of patients with juvenile myoclonic epilepsy. Coordinates of fMRI activation differences are provided in MNI space. In table entries for a given set of MNI coordinates, statistical details *on the left* of the vertical divider (|) refer to the results of the main analyses, and correspond to those reported in Tables S2-S4, while statistical details *on the right* refer to sensitivity analyses controlling for time of day of MRI acquisition. All *P*-values, relating to differences in motor system activation (precentral gyrus, supplementary motor area), are family-wise error rate (FWE) corrected for multiple comparisons within small volume, using a 12-mm diameter sphere centred on local maxima.

Z-scores and *P-*values in brackets refer to repeat group analyses including age, sex and handedness as regressors of no interest, which produced overlapping results. As clarified above, statistical details *on the left* of the vertical divider (|) refer to analyses not controlling for time of day, and correspond to those reported in Tables S2-S4, while statistical details *on the right* refer to analyses additional controlling for time of day, in addition to age, sex and handedness.
