# Supplementary material for: Motor hyperactivation during cognitive tasks: An endophenotype of juvenile myoclonic epilepsy
Source: Epilepsia. 2020 Jun 25;61(7):1438–52. doi: 10.1111/epi.16575 (PMC7681252; doi:10.1111/epi.16575)
Supplement: Supplementary file 6 — Appendix S1 [file EPI-61-1438-s006.docx]

**Appendix 1.**

***S1.1*** *Patients with JME: further clinical details*

History of febrile seizures was documented for 5 patients with JME (16%), not for siblings or controls. 53% of patients had been seizure free for longer than a year. Among patients with ongoing seizures, myoclonic jerks were reported by 87% [13/15; median monthly frequency (IQR): 4.5 (14.1); frequency not quantifiable in 3 subjects], generalized tonic-clonic seizures by 73% [11/15; median monthly frequency (IQR): 0.4 (0.8)], and absence seizures by 87% [13/15; median monthly frequency (IQR): 2.5 (14.7); frequency not quantifiable in 3 subjects]. The median number of anti-epileptic drugs (AEDs) received at time of scan (including p.r.n. benzodiazepines) was 2 (1), with the most frequently prescribed AED being sodium valproate (72% of patients; details in Table S1). The median number of AEDs trialed since disease onset was 3 (2).

***S1.2*** *Neuropsychological test details*

Verbal fluency was assessed with the Controlled Oral Word Association Test, for letter fluency, and with a category fluency test. Letter fluency scores represent the sum of words generated for letter “F”, “A” and “S”; category fluency scores are the sum of items generated for the categories “Animals”, “Fruits”’ and “Vegetables”.^e1^ Memory was assessed using the List and Design Learning subtests from the Adult Memory and Information Processing Battery, and the number of correct responses over the five learning trials (A1 to A5), and on the delayed recall trial (A6), were the scores employed.^e2^ Handedness was determined via the Edinburgh Handedness Inventory. The Hospital Anxiety and Depression Scale measured mood.^e3^ The Trail Making Test (A and B-A) provided measures of psychomotor speed and mental flexibility, respectively,^e4^ while the Digit Span and Arithmetic subtests of the Wechsler Adult Intelligence Scale III assessed working memory. Neuropsychological test scores are reported as raw.

***S1.3*** *Functional MRI data: imaging sequence and preprocessing details*

Functional MRI data was acquired using a 50-slice gradient echo-planar sequence, 64x64 matrix, voxel size 3.75x3.75mm, 2.4mm slice thickness, 0.1mm inter-slice gap, echo time/repetition time: 25/2500ms, flip angle: 70°. For both imaging fMRI tasks, Statistical Parametric Mapping 8 (version 5236) was used for image processing. Functional time series were realigned for motion correction, normalized to a scanner- and acquisition-specific echo-planar imaging template in Montreal Neurological Institute (MNI) space, resampled to isotropic 3x3x3mm voxels, and smoothed with a Gaussian kernel of 8mm full-width at half-maximum.

***S1.4*** *Effects of handedness: supplementary analyses*

Across tasks, repeat group comparisons including age, sex and handedness as covariates (Main Text, Sections 3.3, 3.4, 3.5; Tables S2-S4) produced virtually identical results to our main analyses, pointing to non-significant effects of handedness on intergroup differences.

To further explore the effects of handedness on motor system activation across groups, we also report standardized regression (β) coefficients for the factor “handedness”, that were obtained during adjustment for demographic variables (age/sex/handedness) of individual-level contrast estimates of motor activation for areas of common intergroup differences (these metrics of motor system activation were those employed for ROC curve analyses; extraction procedures are detailed in the Main Text, Section 2.6). For the effect of handedness, we found: *β*=0.14, *t*=1.05, *p*=0.30, for motor activity during memory, left precentral location; *β*=0.003, *t*=0.2, p=0.98 , for motor activity during language, left precentral location; *β* =0.11, *t*=0.82, *p*=0.42 for motor activity across all fMRI task conditions, left precentral location).

Moreover, we further explored motor effects in individuals with atypical handedness (n=5, left-handed; 2 JME patients, 1 sibling, 2 controls). In view of the very small number of subjects, group comparisons were impractical; individual-level data were instead examined. Importantly, none of these subjects appeared as an outlier for motor system effects in any of the tasks. Percentiles for activation measures in each of these subjects with left handedness are reported in the Table at the end of this section. Across tasks and groups, overall no systematic relation emerges: one control fell at the low-end of data distribution in their respective groups, one JME had within-group placements at opposite ends for memory and language, the sibling clustered around the 20^th^ percentile, one control exhibited median values, one JME patients consistently fell at the higher end of the distribution.

| **Subject** | ***Motor Activation - Memory*** | ***Motor Activation - Language*** | ***Motor Activation –***  ***Combined*** |
| --- | --- | --- | --- |
| JME_0013 | 89^th^ JME percentile | 87^th^ JME percentile | 96^th^ JME percentile |
| JME_0025 | 4^th^ JME percentile | 96^th^ JME percentile | 7^th^ JME percentile |
| SIB_0012 | 25^th^ SIB percentile | 33^rd^ SIB percentile | 17^th^ SIB percentile |
| CTR_0024 | 5^th^ CTR percentile | 16^th^ CTR percentile | 6^th^ CTR percentile |
| CTR_0025 | 50^th^ CTR percentile | 44^th^ CTR percentile | 42^nd^ CTR percentile |

***S1.5*** *Laterality indices for verbal processing: supplementary analyses*

*Post-hoc* Spearman correlation analyses probed potential associations between frontal LIs for verbal processing and age-, sex- and handedness-adjusted individual contrast estimates of motor system activation (*β* weights), derived from areas of common intergroup differences (same estimates used for ROC curve analyses; extraction described in the Main Text, Section 2.6). There were no significant correlations between frontal language LI and estimates of motor activation during language (*ρ=*-0.10*, p=*0.40), nor between frontal verbal memory LI and estimates of motor activation during memory (*ρ=*0.12*, p=*0.36). Partial Spearman correlations, controlling for group allocation, remained non-significant (partial *ρ=*-0.14*, p=*0.26 for language*;* partial *ρ=*0.09*, p=*0.49 for memory). These analyses rule out a potential modulation of motor system activation by patterns of verbal hemispheric dominance.

***S1.6*** *Motor system laterality indices across all task conditions: supplementary analyses*

*Post-hoc* Spearman correlation analyses probed potential associations between (1) LIs of motor system activation, based on a bilateral mask comprising precentral gyri and bilateral supplementary motor areas, and (2) and age-, sex- and handedness-adjusted individual contrast estimates of motor system activation (β weights) derived from areas of common intergroup differences (same estimates used for ROC curve analyses; extraction described in the Main Text, Section 2.6). There were no significant correlations between motor system LIs and motor activation during language conditions, with or without controlling for group allocation (*ρ/partial ρ=*-0.20/-0.13*, p=*0.11/0.29, “Repeat” condition; *ρ/partial ρ=*-0.003/-0.03*, p=*0.98/0.79, “Generate” condition), nor between motor system LI and motor activation during memory conditions (*ρ/partial ρ=*-0.05/0.01*, p=*0.73/0.96, “Pictures”; *ρ/partial ρ=*-0.07/-0.03*, p=*0.58/0.81, “Words”; *ρ/partial ρ=*0.14/0.20*, p=*0.30/0.14, “Faces”). Collectively, these analyses rule out a significant modulation of motor system activation by laterality profiles of motor system activity during all task conditions.

***S1.7*** *Post-hoc correlations: left precentral activation and time since last seizure*

We conducted exploratory Spearman’s rank correlations between (a) “time since last seizure” and (b) age-, sex- and handedness-adjusted individual contrast estimates of motor activation, extracted from left precentral coordinates of maximum intergroup differences between JME with and without ongoing seizures, for language, memory and the combined task; MNI coordinates for extraction of individual level contrast estimates were: x=-60, y=-4, z=40, for memory fMRI; x=-39, y=-10, z=67, for language fMRI; x=-33, y=-10, z=61 for the combined fMRI model. There were significant associations between time since last seizure and left precentral activation during memory and the combined fMRI task, with shorter intervals since last seizure associated with stronger activation (*ρ=*-0.39*, p=*0.04, memory fMRI; *ρ=*-0.55*, p=*0.005, combined fMRI model). Correlations were also negative for motor activation during language fMRI, but did not reach significance (*ρ=*-0.22*, p=*0.24).

***S1.8*** *Sensitivity analyses: influence of time of MRI acquisition*

1) *Correlations between time of MRI acquisition and metrics of motor system activation*. Across all participants, *post-hoc* Spearman’s rank correlation analyses tested for associations between time of day (hour) and parameter estimates of motor system activation. As a marker of the latter, we employed the same metrics used for ROC curve analyses, *i.e.* age-, sex- and handedness-adjusted parameter estimates of motor system activation extracted from areas of common intergroup differences (extraction detailed in section 3.5, main text). We found non-significant associations between time of day and motor system activation for memory (*ρ*= 0.14, *p*=0.29), language (*ρ*=0.08, *p=*0.51) and the composite model across tasks (*ρ*=0.15, *p*=0.28). Partial Spearman’s rank correlations, controlling for group allocation, remained non-significant (partial *ρ*=0.19, *p*=0.16; partial *ρ*=0.11, *p*=0.38; partial *ρ*=0.21, *p*=0.12 for memory, language and the composite model, respectively);

2) *Repeat second-level analyses for comparisons involving groups with significant or near-significant differences.* We repeated second-level imaging analyses for comparisons involving: (a) siblings against controls and (b) conjunction analyses, which focus on all three study groups. Results are reported in Table S5, where where we juxtapose (a) findings of our main analyses, comparing groups with and without age, sex and handedness as covariates (reported in Table S2/S3/S4 for memory fMRI/language fMRI/combined fMRI model), with (b) results of repeat comparisons, employing time of day as nuisance regressor in all models. Both motor as well as whole-brain comparisons of effects remained substantially unchanged, demonstrating a marginal influence of time of day on group-level differences;

3) *Repeat ROC curve analyses for discrimination of a combined JME-sibling group from controls.* As detailed in paragraph 3.5 of the main text, we devised ROC curve analyses to explore discrimination from patients with JME and a combined patient-sibling group from healthy controls via parameter estimates of motor activation, extracted from areas of common intergroup differences. To investigate the potential influence of time of MRI acquisition, we repeated these ROC curve analyses employing age-, sex-, handedness- as well as time-of-day-adjusted parameter estimates of motor activation for both tasks and the composite model. Results of these repeat ROC curve models were virtually identical to those of the main analysis [memory fMRI: previous/time-adjusted AUC= 0.74/0.74, SE=0.07/0.07, p=0.003/0.002; language fMRI: previous/time-adjusted AUC=0.69/0.70, SE=0.07/0.07, p=0.009/0.007; composite marker of motor activity across tasks: AUC=0.77/0.75, SE=0.06/0.07, p=0.001/0.002], substantiating a small, negligible influence of time of MRI acquisition on subgroup discrimination.

***S1.9*** *Supplementary References*

1. Bird CM, Papadopoulou K, Ricciardelli P, Rossor MN, Cipolotti L. Monitoring cognitive changes: psychometric properties of six cognitive tests Br J Clin Psychol. 2004 Jun;43:197-210.

2. Coughlan AK, Hollows SE. The adult memory and information processing battery (AMIPB) Psychology Department, St James Hospital, Leeds, UK. 1985.

3. Zigmond AS, Snaith RP. The hospital anxiety and depression scale Acta Psychiatr Scand. 1983 Jun;67:361-370.

4. Tombaugh TN. Trail Making Test A and B: normative data stratified by age and education Arch Clin Neuropsychol. 2004 Mar;19:203-214.
